# Less is more: dimensionality reduction as a general strategy for more precise luminescence thermometry

**DOI:** 10.1038/s41377-022-00932-3

**Published:** 2022-07-27

**Authors:** Erving Ximendes, Riccardo Marin, Luis Dias Carlos, Daniel Jaque

**Affiliations:** 1 NanoBIG, Departamento de Fısica de Materiales, Facultad de Ciencias, Universidad Autónoma de Madrid, C/Francisco Tomás y Valiente 7, Madrid, 28049 Spain; 2 NanoBIG, Instituto Ramón y Cajal de Investigación Sanitaria (IRYCIS), Ctra. Colmenar km. 9.100, Madrid, 28034 Spain; 3 Phantom-g, CICECO – Aveiro Institute of Materials, Department of Physics, University of Aveiro, Aveiro, 3810-193 Portugal

**Keywords:** Optics and photonics, Optical materials and structures

## Abstract

Thermal resolution (also referred to as temperature uncertainty) establishes the minimum discernible temperature change sensed by luminescent thermometers and is a key figure of merit to rank them. Much has been done to minimize its value via probe optimization and correction of readout artifacts, but little effort was put into a better exploitation of calibration datasets. In this context, this work aims at providing a new perspective on the definition of luminescence-based thermometric parameters using dimensionality reduction techniques that emerged in the last years. The application of linear (Principal Component Analysis) and non-linear (t-distributed Stochastic Neighbor Embedding) transformations to the calibration datasets obtained from rare-earth nanoparticles and semiconductor nanocrystals resulted in an improvement in thermal resolution compared to the more classical intensity-based and ratiometric approaches. This, in turn, enabled precise monitoring of temperature changes smaller than 0.1 °C. The methods here presented allow choosing superior thermometric parameters compared to the more classical ones, pushing the performance of luminescent thermometers close to the experimentally achievable limits.

## Abstract (web summary)

Dimensionality reduction techniques are applied in luminescence thermometry for more precise readouts.

## Graphical abstract

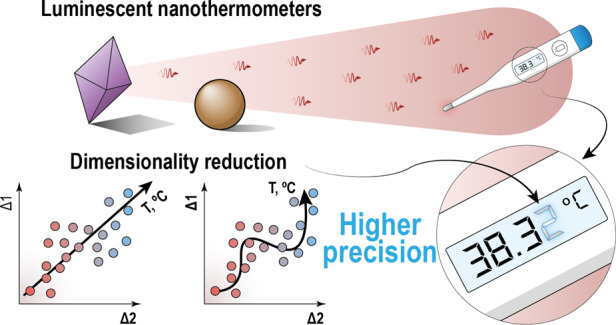

## Introduction

*Much testing; accuracy and precision in experiment; no guesswork or self-deception*. Martha Marquardt, redacting the biography of the German scientist Paul Ehrlich, whom she served as secretary, credits these words to the father of chemotherapy and 1908 Nobel laureate in Physiology or Medicine^1^. This quote, in a sharp yet elegant way, summarizes good practice in scientific investigation. The availability of *accurate* and *precise* tools is key to avoid *self-deception* during experimentation, but it also allows to capture a snapshot of elusive phenomena that could go otherwise unnoticed. Despite its young age, the field of luminescence thermometry is no exception to this pursuit for ever higher precision and accuracy.

In luminescence thermometry, temperature-induced changes in the spectroscopic properties of an ensemble of probes (e.g., dye molecules, metal complexes, or nanoparticles) are harnessed to remotely obtain a thermal readout of the environment with which the probe is in contact. This technology has strong appeal, among other fields, in the biomedical context, as demonstrated by its applications to study intracellular temperature, determine physical properties of biological tissues, and detect diseases under in vivo conditions^2–5^. As it happens with any expanding sphere of knowledge, during the use of this sensing technology some complications started to be observed and solutions began to be proposed^6–8^. For instance, irreproducibility in the synthesis of luminescent species or erroneous readouts caused by photon attenuation induced by biological tissues have been abundantly addressed^9–14^. Another class of conundrums, which pertain to the conceptual level and to data post-processing, have been only pragmatically and less systematically dealt with. As a result, the field of luminescence thermometry is repleted by the day with new luminescent probes but few are the improvements at the methodological level; that is, with the exception of a handful of recent works involving multiparametric readouts and machine learning-based regression models^15–17^. By exploring the use of dimensionality reduction (DR), our work fits in this frame of underexplored data processing methods, catering to researchers whose goal is to maximize the performance of a luminescence thermometry approach.

At the base of the comparison between luminescent thermometers (LThs) are widely accepted figures of merit, which allow us to rank thermometric approaches^18^. Among them, temperature uncertainty (also referred to as thermal resolution), δ*T*, establishes the minimum temperature difference detected by a LTh under specific experimental conditions. Its definition is given by^19^:

$$\delta T = \frac{{\partial T}}{{\partial {{\Delta }}}}\cdot \delta {{\Delta }}$$

where δΔ is the uncertainty in the determination of the thermometric parameter Δ (i.e., the spectroscopic parameter that provides the thermal readout). δ*T* hence estimates the statistical uncertainty (precision) of the thermometric approach of interest. Moreover, it provides a normalized value that can be compared regardless of the nature of the chosen luminescent thermometer and thermometric parameter. It justifies, therefore, its existence as a figure of merit. The researchers are left to investigate and select the most appropriate thermometric parameter that minimizes δ*T*. This can be a relatively simple task for some LThs (e.g., for luminescence lifetime-based LThs), but it is not so straightforward when multiple temperature-dependent spectroscopic parameters are present (i.e., the so-called multiparametric LThs).

Traditionally a trial-and-error approach has been used to identify the parameter that is most sensitive towards temperature. Maturi et al. demonstrated that such plan of action often leads to an underperformance of the thermometer^20^. The authors showed that a more reasonable approach is to select parameters that linearly depend on the temperature and build a multiple linear regression model. This approach constitutes certainly an advancement, yet the human component still heavily governs the decision making in the selection of thermometric parameters to build the linear regression model. Its applicability is also limited to parameters with a linear dependence on temperature. And there is always the possibility of error increment due to the collinearity of dependent variables. A more generally applicable strategy that does not require a preventive identification of specific parameters is currently lacking; a gap that likely cripples the real potential of most proposed thermometric approaches.

*Dimensionality reduction* (DR) methods can help in this context. Simply put, DR is the transformation of data from a high-dimensional space into one with lower dimensionality such that the final representation retains most of its meaningful properties. DR techniques gained momentum in the last couple of years thanks to the generation of high-dimensionality data^21,22^, and are at the basis of applications involving *machine learning* including voice recognition, pattern identification, and noise reduction^23–25^. Because the calibration of a luminescent thermometer generates large datasets (e.g., intensity vs. several wavelengths at different temperatures), extending DR approaches to luminescence thermometry is a natural step to identify the numerical quantities that better correlate with temperature. However, only few examples of DR methods applied to luminescence thermometry have been reported^15–17^, Lewis et al., for instance, obtained a thermal readout through a long short-term memory neural network trained with a combination of raw spectral and time-resolved luminescence data obtained from quantum dots^15^. Šević et al., on the other hand, used principal component analysis to infer the temperature from the luminescence of Sr_2_CeO_4_:Eu^3+^ nanophosphors^17^. Both studies, however, built regression models based on a single calibration dataset. Thus, thermal resolution or repeatability could not be determined, preventing quantitative assessment of the complete readout improvement through figures of merit.

In this work, we show how DR can enhance the performance of luminescence thermometry based on near-infrared (NIR) emitting rare-earth nanoparticles (RENPs) in the biological temperature range (31–45 °C). Specifically, we show that linear and non-linear DR methods improve the precision of the thermometric approach compared to a classical ratiometric approach. Extension of the data treatment to datasets gathered for the temperature-dependent NIR emission of Ag_2_S semiconductor nanocrystals (SNCs) confirms the general applicability of the proposed methods.

## Results

### Nanoparticle selection

**Fig. 1:**
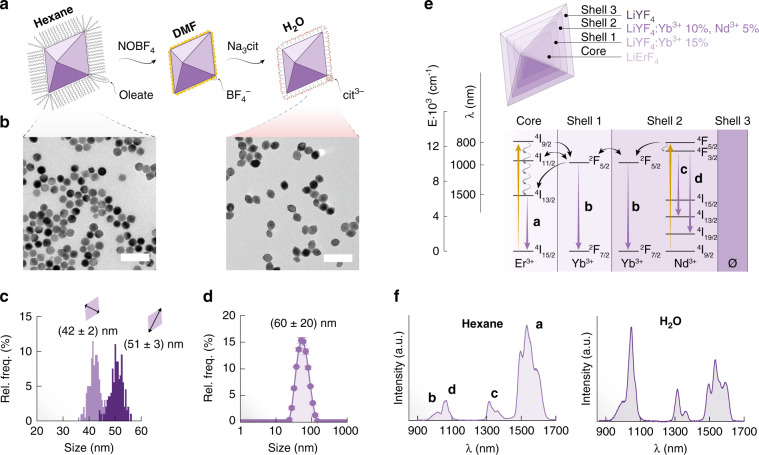
The selected RENPs. **a** Scheme of the steps followed to transfer the as-synthesized hydrophobic RENPs to water. DMF = *N,N*-dimethylformamide and cit^3–^ = citrate ion. **b** Image obtained with a transmission electron microscope (TEM) of the as-synthesized (left) and water-dispersed (right) core/shell/shell/shell RENPs along with **c** a size distribution histogram for the minor and major axes of the bipyramidal RENPs and **d** hydrodynamic size distribution derived from dynamic light scattering measurements. TEM scale bars are 200 nm. **e** Scheme showing the architecture and composition of the various volumes of a RENP along with partial energy level scheme of the doping rare-earth ions. Absorption processes under 790 nm excitation are depicted as vertical yellow arrows, emissions as vertical purple arrows, ET as black curvy arrows, and non-radiative relaxation phenomena as squiggly gray arrows. **f** Comparison of the NIR emission under 790-nm excitation of the RENPs in hexane (left) and after transfer to water (right)

The water-dispersible RENPs acting as luminescent thermometers (Fig. 1a–d) were purposely designed to maximize the number of temperature-dependent variables at play (Fig. 1e, f). These include variations of relative intensity between transitions arising from different ions and/or within a single manifold (i.e., changes in the relative intensity of Stark components), as well as possible spectral shifts. This is a challenging situation for the selection of the most sensitive (hence most precise) thermometric parameter in a classical way since several changes occur simultaneously. Even an experienced researcher in the field might struggle to select the best integration intervals and/or properly deconvolute the signals, let alone choosing a combination thereof. The RENPs were prepared adapting a previously developed approach (see details in Supporting Information) and have a core/shell/shell/shell architecture (Fig. 1e) that aims at covering the broadest NIR wavelength range (Fig. 1f, S1)^26^.

Preference was given to a NIR-emitting system because of the relevance of this wavelength range for biomedical applications^27^. Signals arising from Er^3+^, Yb^3+^, and Nd^3+^ were observed throughout the whole 850–1600 nm range under 790-nm excitation. These signals arise from direct excitation of Er^3+^ and Nd^3+^ as well as energy transfer (ET) processes occurring between ions (Fig. 1e). Aside from NIR downshifting emission, UC photoluminescence under the same excitation wavelength was observed (Figure S2). The RENPs have a bipyramidal morphology (Fig. 1b), with characteristic sizes of (42 ± 2) and (51 ± 3) nm for their minor and major axis, respectively (Fig. 1c). Since the as-prepared RENPs are hydrophobic, they were transferred to distilled water by NOBF_4_-mediated removal of oleic acid molecules coordinated to the RENPs surface, followed by decoration with citrate molecules (Fig. 1a, b). Citrate molecules imparted colloidal stability to the individually dispersed RENPs (Fig. 1d). As expected, the relative intensity of the emission bands arising from the different rare earth ions changes sizably when passing from an organic solvent (here hexanes) to water (Fig. 1f). This is a consequence of the different vibrational energy featured by the solvent molecules, which has also been shown to determine the thermometric performance of RENPs^28^. Note that no optimization of the composition of the different shell was attempted, given that the goal of this study was not to obtain the best performing system.

### Experiment

**Fig. 2:**
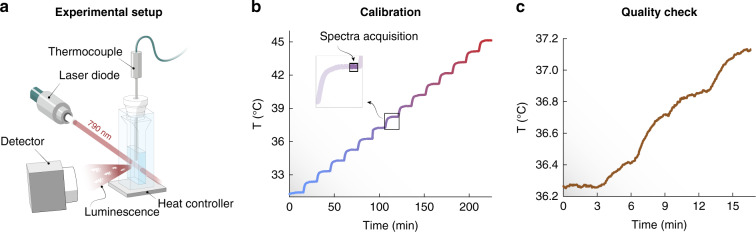
Thermometer calibration and validation. **a** Schematic representation of the experimental setup. A cuvette filled with a dispersion of RENPs is excited by a 790 nm laser-diode and its temperature—which is varied in a controlled way externally—is monitored with a thermocouple. **b** Temperature obtained with the thermocouple (and taken as the gold standard) during the calibration of the luminescent thermometer. **c** Temperature profile obtained with thermocouple for quality-check

To determine the behavior of the RENPs under temperature variations, the experimental setup depicted in Fig. 2a was used (see Materials & Methods for further details). It entails the use of a quartz cuvette filled with an aqueous dispersion of the RENPs placed in a sample holder where the temperature can be externally controlled. A thermocouple was inserted in the cuvette, with its tip immersed in the RENP dispersion. A 790 nm laser diode was used to excite the RENPs just below the tip of the thermocouple. The proximity of the tip to the laser path minimizes discrepancies between the actual temperature of the dispersion in correspondence of the laser path and the one picked up by the thermocouple. The power density on the sample was kept at a value that allowed the minimization of heating induced by photothermal effects (2.5–3.0 W·cm^−2^, see Table 1).

**Table 1:** Summary of the experimental conditions and relevant parameters for each of the three sets of measurements

Sample	Exposure time [s]	Slit width [μm]	Beam area [cm^2^]	Power density [W cm^−2^]	SNR at 31 °C	SNR at 45 °C	Heating when laser on [°C]
RENPs	3	200	4.9·10^−2^	2.8	230-77	225-76	0.08
Ag_2_S SNCs	2	200	4.9·10^−2^	1	83	8	N/A

For RENPs, the two SNRs values for each temperature are extracted for the strongest Nd^3+^ emission and Er^3+^ emission, respectively

The first part of the experiment consisted in calibrating the thermometers measuring their fluorescence signal as a function of temperature. Thus, with the aid of the heat controller, the temperature of the system was varied from 31 to 45 °C with a step of 1 °C (Fig. 2b). In each of the 15 steps, 100 luminescence spectra were recorded after the temperature stabilized—i.e., when fluctuations were smaller than 0.05 °C. The representative time interval in which these acquisitions took place is shown in the inset of Fig. 2b. For each of the 15 temperatures, 100 values of the elected thermometric parameter were thus obtained along with a meaningful estimation of the associated intrinsic error (taken here as the standard deviation of the parameter). Note that this large dataset (1500 spectra in total, along the path of *much testing* mentioned above) is also necessary to reliably employ the DR approaches. Such a large dataset, in turn, is important in the estimation of the thermal resolution.

The second part of the experiment aimed at comparing the quality of the thermal reading provided by the chosen thermometric parameters. To do so, a new set of data was obtained varying the temperature of the system from 36.2 to 37.2 °C with a step size five times smaller than the one used during the calibration (i.e., 0.2 °C). Note that the reliability of luminescent thermometers is not generally tested with such small temperature increments. During the whole process, both the reading from the thermocouple (Fig. 2c) and the luminescence signal were recorded for comparison. The rationale being that the parameters with the best performance would better resemble the reading provided by the thermocouple in a *precise* fashion.

### Comparison of the approaches

#### Classical approach

**Fig. 3:**
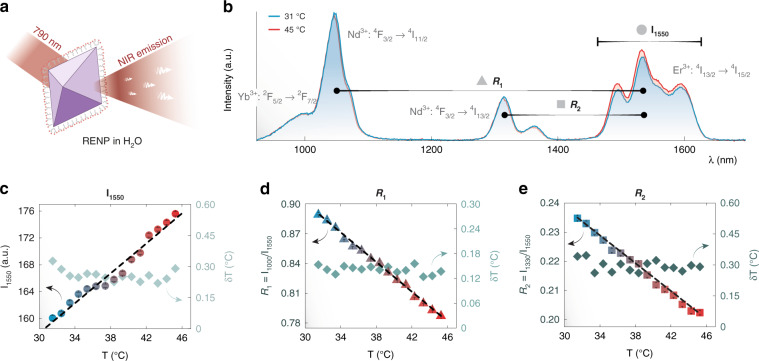
The classical approach for the identification of thermometric parameters. **a** Sketch of one multishell RENP coated with citrate ions, dispersed in water, being excited with a 790 nm laser and emitting NIR luminescence. **b** Luminescence spectra (averaged over the 100 measurements) of RENPs under 790-nm excitation obtained at 31 and 45 °C. An increment in the Er^3+^ emission band centered at 1550 nm (Er^3+^: ^4^I_13/2_ → ^4^I_15/2_) is observed. Thermal dependence of **c** the integrated emission of Er^3+^: ^4^I_13/2_ → ^4^I_15/2_ (I_1550_), **d** the ratio between the integrated emissions of (i) Yb^3+^: ^2^F_5/2_ → ^2^F_7/2_ and Nd^3+^: ^4^F_3 /2_ → ^4^I_11/2_ and (ii) Er^3+^: ^4^I_13/2_ → ^4^I_15/2_ (**R**_**1**_), and **e** the ratio between the integrated emissions of Nd^3+^: ^4^F_3/2_ → ^4^I_13/2_, and Er^3+^: ^4^I_13/2_ → ^4^I_15/2_ (**R**_**2**_) alongside with their thermal resolutions. Error bars are standard deviations. The right vertical axis of (c) ranged from 0 to 0.3 for further comparison with DR-based parameters. Dash black lines are linear fits to the experimental data. For the three parameters, *R*^*2*^ values are 0.965 (I_1550_), 0.996 (R_1_), and 0.994 (R_2_) respectively

The thermal response of the RENPs used in this work is summarized in Fig. 3, which contains a scheme of the RENPs (Fig. 3a) along with the average luminescence spectra corresponding to 31 and 45 °C (Fig. 3b). As expected, the sample presents a sensitivity of the same order of magnitude of those generally reported for rare-earth doped NPs (which is much lower than, e.g., Ag_2_S SNCs with emission in the NIR)^29,30^. Indeed, in our data, only a slight increment in the intensity of the Er^3+^ emission band centered at 1550 nm is observed over the tested temperature range—a behavior similar to the one previously observed in Er^3+^-containing NIR luminescent thermometers^29^. Thus, following the classical route of (informed) *guesswork* to select the thermometric parameter, there are three reasonable options: (i) the integrated emission, I_1550_, of Er^3+^, (ii) the luminescence intensity ratio, R_1_, between the integrated emissions of Yb^3+^ + Nd^3+^ around 1000 nm and the 1550 nm emission band of Er^3+^, or (iii) the ratio, R_2_, between the integrated emissions of the 1330 nm band of Nd^3+^ and the 1550 nm band of Er^3+^. By calculating the average value and standard deviation of these parameters from the 100 measurements at each step of the calibration, one obtains the graphs in Fig. 3c–e. The histogram plots of the values assumed by I_1550_, R_1_ and R_2_ during calibration are included in Figure S3 for further inspection. All three parameters have a linear dependence with temperature, with slopes of 1.10 a.u.·°C^−1^, −0.00747 °C^−1^ and −0.00243 °C^−1^ for I_1550_, R_1_ and R_2,_ respectively. The corresponding relative thermal sensitivities (*S*_*r*_) were 0.6, 0.9 and 1.1% °C^−1^. These values are comparable with those of other RENPs working in the NIR (Table S1).

Despite the similar linear behavior and relative sensitivities, some of these parameters were associated with higher uncertainty than others (as highlighted by the difference in broadness of the histogram distributions in Fig. S3). The thermal uncertainties of the three thermometric parameters (I_1550_, R_1_, and R_2_) were calculated (Fig. 2b−d, right *y*-axes) and, evidently, in the 31–45 °C range they did not provide an average temperature uncertainty lower than 0.15 °C. This quantity will be treated as a reference for the output of the different DR approaches. Note that while R_2_ had a higher relative thermal sensitivity, it was accompanied by a higher uncertainty (in the order of 0.3 °C). The reason being that R_2_ had a lower signal-to-noise ratio than R_1_. This result supports the observation that searching for parameters with higher sensitivity may not always lead to better thermal readouts^31,32^.

#### DR-based approach

**Fig. 4:**
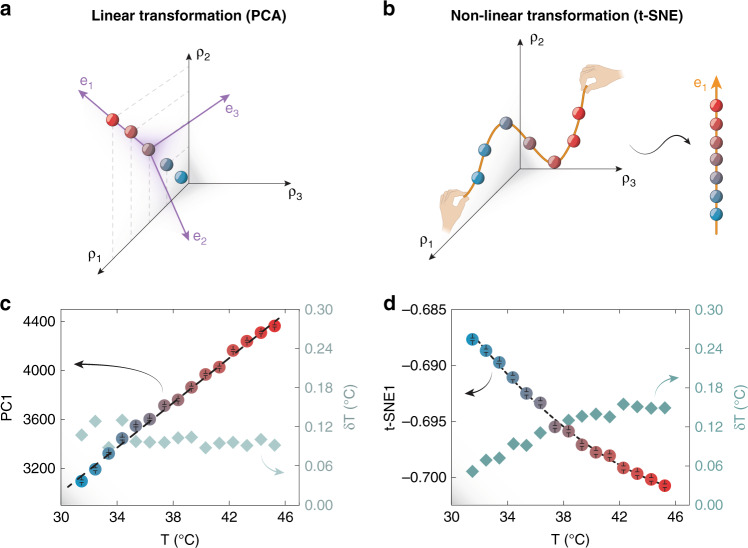
Linear and non-linear dimensionality reduction in luminescence thermometry. **a** When applying linear transformations to the input data, a new basis is found (purple axes) for the linear space and new coordinates are given to the data. These coordinates are organized according to their level of importance. **b** Non-linear transformations look, after considering the geodesic defined by the data, for the shortest path between all combination of points and try to unroll it into a space of smaller dimensions. In the graph, this is exemplified by a curvy thread (which needed three dimensions to be described) being stretched out into a straight line (only one dimension). In this work, the algorithms of PCA and t-SNE were used as representative linear and non-linear transformations, respectively. **c** Dependence of the first principal component, PC1, of the calibration dataset with temperature. A quasi-linear relationship is observed and an average uncertainty of 0.09 °C in the 31–45 °C range (represented as blue diamonds) is achieved. **d** Thermal dependence of the first coordinate of the t-SNE transformation of the calibration dataset. A parabolic relationship is observed and an average uncertainty of 0.13 °C in the 31–45 °C range (represented as blue diamonds) is achieved. Dashed black lines are linear (c, *R*^*2*^ = 0.995) and parabolic (d, *R*^*2*^ = 0.996) fits to the experimental data

Before we discuss the application of DR to the dataset in Fig. 3b, it is worth briefly mentioning the main methods one could use. DR could proceed through (i) the removal of redundant input features, which is generally achieved by setting up a correlation matrix and verifying the absolute values of off-diagonal entries which are closer to the unity; or (ii) by the transformation of the input data, which has its focus on presenting the information with more recognizable patterns^21^. A linear DR method entails a transformation where a linear combination of the original variables is sought after (Fig. 4a). The well-known algorithms of principal component analysis (PCA) and multidimensional scaling (MDS) belong to the linear class^33^. In the category of non-linear transformation-based DR approaches fall instead t-distributed stochastic neighbor embedding (t-SNE), locally linear embedding (LLE) and isometric mapping (Isomap)^33,34^. For the sake of our discussion, we will focus our attention on PCA and t-SNE for two reasons: (i) they are, respectively, good representatives of DR approaches based on linear and non-linear transformations of the input data and (ii) they do not require a high density of data points to perform well. The latter point is important because in a calibration one usually selects a well-defined step to move from one temperature to another. The spectra corresponding to in-between temperatures, however, are not measured. Thus, if the method is extremely dependent on a high density of data points (as is the case for LLE and Isomap), the projection of the dataset onto a space of lower dimension may not work well in these smaller ranges of temperature.

To give a detailed account of PCA and t-SNE, we refer the reader to the Materials & Methods Section, where their algorithms are described. A more in-depth description of how they work can be found in published literature^33,35^. We nonetheless present here general statements about their operative principles. In general, the collected *p*-dimensional dataset *x*_*j*_ (*j* = 1 … *p*) is mutually correlated and the datapoints form a *p*-dimensional ellipsoid. PCA allows to represent the collected dataset in a diagonalized basis set. The respective mutually orthogonal *p* eigenvectors are the different principal axes (i.e., principal components) of the ellipsoid and represent a measure for the variances along the different directions *x*_*j*_. This is the most convenient basis to describe the dataset. In t-SNE, on the other hand, the concept is to assume a model probability distribution of the dataset and then verify how much a measured data point deviates from the data point expected according to the model of probability distribution. This difference is the Kullback-Leibler divergence and must be minimized.

In our system, after applying PCA to the calibration dataset, one finds that 22 variables (principal components) are needed to account for 90% of the variance of the original data (Fig. S5). The high number of PCs needed is justified by the insertion of the first-order derivative of the spectra as input for the DR routine—an inclusion motivated by its power to correct for background effects on the signal^36^. However, to have a one-to-one comparison with the previous parameters, we decided to proceed with the analysis using only the first principal component (PC1). In addition, while the other PCs could have been considered for a multiple regression model, the fact that they did not present an unequivocal relationship with temperature (i.e., they are not invertible) could narrow the temperature range in which the model works well (Fig. S6). PC1, on the other hand, was invertible with temperature in the whole range in which the calibration was measured and alone accounted for the majority (63%) of the variance of the dataset. When projecting the calibration dataset into the direction of this component, one finds the behavior depicted in Fig. 4c. A quasi-linear dependence of PC1 with the temperature is observed. By considering the intrinsic error in its determination, an average thermal uncertainty of 0.09 °C is achieved (right axis of Fig. 4c).

On the other hand, when applying t-SNE to the calibration data, a careful balance between the attention given to the local and global aspects of the calibration needs to be achieved. This is done by carefully tuning the value of a free parameter called *perplexity*. Such parameter is a measure of information and can be viewed as a knob that sets the number of effective nearest neighbors^37^. As a general rule of thumb, one can assume that the perplexity should be of the order of the square root of the total number of measurements (in our calibration, $\sqrt {1500} \approx 39$)^34^. In our case, however, we have discovered that a slightly lower perplexity of 35 yields a better clustering of the points corresponding to the 15 temperatures. When applying the t-SNE algorithm to our experimental dataset with an output dimension of 2, the dependence with temperature of the first coordinate (t-SNE1) of the low dimensional dataset found in Fig. 4d is observed. For the dependence of the second coordinate see Fig. S7. A parabolic behavior was observed and, similarly, by considering the standard deviation of the parameter t-SNE1, the thermal uncertainty was evaluated (right axis of Fig. 4d). In this case, it seemed to deviate from a constant value, increasing from 0.05 °C to 0.15 °C in the 31–45 °C range. Yet, upon comparison with the thermometric parameters chosen by visual inspection (I_1550_, R_1_ and R_2_), the first coordinate of both PCA (PC1) and t-SNE (t-SNE1) performed better in terms of thermal resolution in the explored temperature range. The histogram plots of the values assumed by PC1 and t-SNE1 during calibration were also included in Fig. S4 for further inspection.

#### Quality-check

**Fig. 5:**
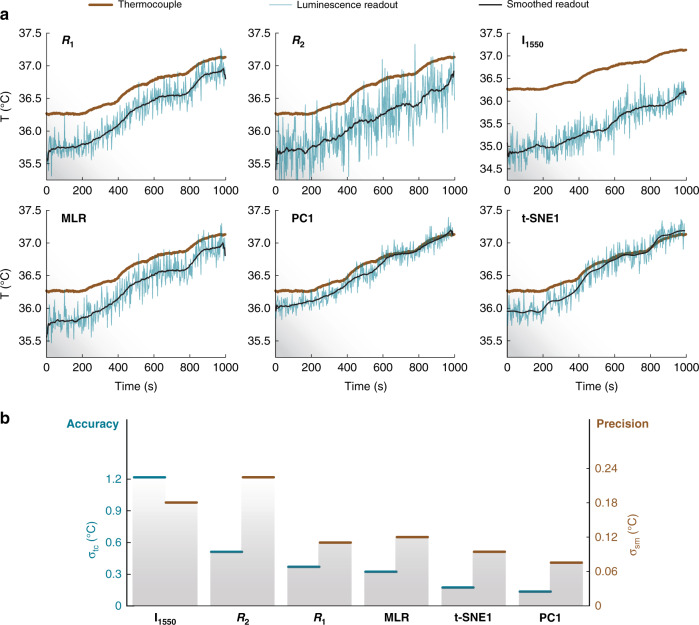
Quality check of readouts provided by different thermometric parameters. **a** Readouts provided by I_1500_, R_1_, R_2_, MLR, PC1, and t-SNE1 during the quality check experiment. A lower noise is observed for the DR-based approaches, even compared to MLR. The black lines stand are the smoothed curves of each readout, as obtained by a Savitzky-Golay filter with 40-points window. **b** Comparison between accuracy and precision of the readouts provided by all the thermometric parameters studied in this work

While DR-based approaches yield more precise calibrations, their true impact on thermal sensing might be more easily verified with a *quality check* experiment, wherein changes as small as 0.2 °C take place (Fig. 2c). To that end, we compared the readings provided by the parameters chosen with the usual approach (I_1500_, R_1_, and R_2_), the two DR-based parameters (PC1 and t-SNE1), and the one retrieved using the multi linear regression approach proposed by Maturi et al. (hereafter MLR, Fig. 5) obtained using a combination of I_1500_, R_1_, and R_2_.

The smoothed curves of each readout – obtained by applying a Savitzky-Golay filter with a 40-points window—are included as black lines to guide the eyes of the reader. In accordance with our expectations, the reading from PC1 and t-SNE1 were less noisy than the ones from I_1500_, R_1_, and R_2_. This demonstrates that the improvement in terms of uncertainty is factual and not just an accident of approximations in the calculations and/or model fitting. It should be noted that, in contrast, the MLR approach does not lead to a noticeable improvement of the precision in this case. The likely reasons being (i) the underlying assumption in MLR that I_1500_, R_1_, and R_2_ are the best parameters to summarize the thermal dependence of the RENPs, and (ii) the error inflation caused by the collinearity of these parameters. The latter issue is a well-documented problem in statistical literature^38^. DR techniques, however, have recently been shown to be effective in solving it^39,40^.

Another aspect of the thermal readout which the DR-based approach improved was the proximity to the value provided by the thermocouple (taken as the gold standard). This is especially curious if one compares the goodness of the linear fits of the calibration datasets found in Figs. 3c and 4c. If one had to judge the accuracy of R_1_ and PC1 based on how close these lines were to the points in the 36–37 °C range, then R_1_ should be the most accurate parameter. However, when put to the test with extra data (Fig. 5), that was not found to be the case. The reading from R_1_ provided an average deviation of 0.36 °C. The one of PC1, however, provided 0.13 °C: a 3-fold enhancement in accuracy. Thus, to summarize the improvements observed so far, we calculated two new quantities for each parameter: (i) the standard deviations of its thermal readout from its smoothed curve, σ_sm_ and (ii) the standard deviations of its thermal readout from the one provided by the thermocouple, σ_tc_. They are, respectively, indicators of the precision and accuracy of the thermal reading. The results are included in Fig. 5b, and they reveal that the approaches based on DR minimize both quantities in these experimental conditions.

At this point, one could wonder if DR-based approaches affect the repeatability of the thermometric approach. In Section S6 (Fig. S8) it is demonstrated that there is little to no effect to this figure of merit. The reason being that the lack of hysteresis is a pre-requisite to apply DR to luminescence thermometry. If this condition is respected, there is little room for improvement in the value of the repeatability.

### Extension to other luminescent thermometers

**Fig. 6:**
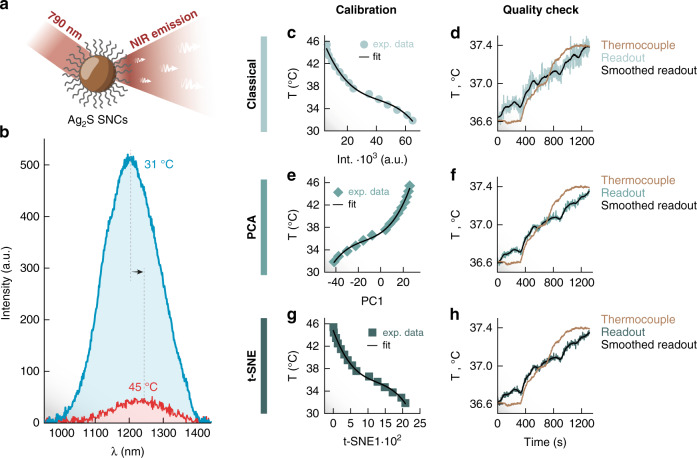
Results for Ag_2_S SNCs. **a** Sketch of the commercial polyethylene glycol-coated Ag_2_S SNCs (SINANO©) used in this study. **b** Photoluminescence spectra under 790 nm excitation recorded at 31 (blue) and 45 °C (red). Results of the calibration and quality check for a classical intensity-based thermometric approach (**c**, **d**), along with the results for luminescence thermometry applying PCA (**e**, **f**) and t-SNE (**g**, **h**) DR methods. For the DR-based methods, a lower noise is observed in the thermal readout. For the three parameters, *R*^*2*^ values are 0.993 (classical), 0.996 (PC_1_), and 0.995 (tSNE_1_) respectively

To demonstrate how DR-based approaches could work for other types of luminescent thermometers, we have repeated the experiments described in the previous sections using Ag_2_S SNCs (Fig. 6a). To increase the level of challenge, we performed them under a regime of low excitation intensity that results in a low overall signal intensity. Such conditions are particularly relevant for biomedical applications where, to avoid damage, the excitation power density needs to stay below specific safety levels. Moreover, photon extinction by biological tissues strongly reduces both the number of excitation photons reaching the luminescent probe (nanoparticle) and the number of emitted photons collected by the detection systems^41^. Swieten et al. have demonstrated that when working in conditions of low signal-to-noise ratio, the thermal uncertainty is affected and the performance of the thermometer is compromised^42^. It is therefore desirable to improve the quality of the thermal readout in such circumstances. Figure 6b contains the luminescence spectra (averaged over the 100 measurements) of Ag_2_S SNCs at 31 and 45 °C. Therein, the level of noise in the spectra is much more noticeable than in the case of RENPs (Fig. 3b). When performing thermometry with the usual parameters (i.e., integrated intensity, peak position, and intensity ratio), one finds that the most precise one is the integrated intensity I_1000-1400_ which provides an uncertainty of 0.13 °C (Figs. S9, S10). Its calibration shows a non-linear dependence with temperature (Fig. 6c) which can be fitted to a third-order polynomial.

**Fig. 7:**
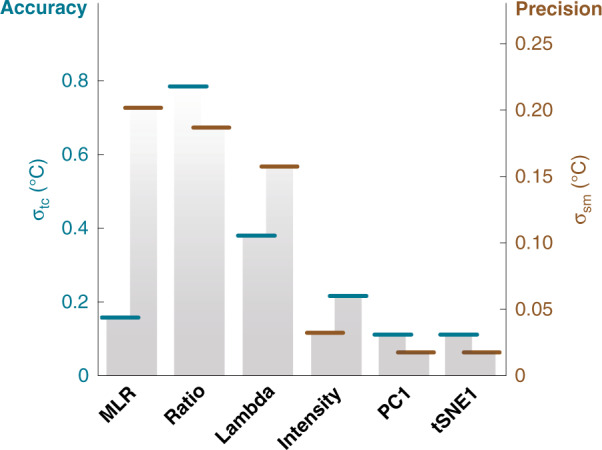
Comparison of accuracy and precision in quality check for Ag_2_S SNCs

When applying the DR approaches to the calibration dataset, non-linear thermal dependences of the new variables, PC1 and t-SNE1, are also observed. Similar to I_1000-1400_, their thermal dependences were fitted to third-order polynomials. However, in these cases, they both provided average uncertainties of 0.06 °C in the 31–45 °C range (Fig. S10). When compared to the usual intensity-based approach, such values constitute a 2-fold improvement in the precision of the readout. This betterment of the performance can be verified by comparing the readouts provided by the classical thermometric parameter (I_1000-1400_) with the ones of PC1 and t-SNE1 during the quality check (Fig. 6d, f, h). As illustrated in Fig. 7, although the accuracy remains substantially unvaried, the fluctuations of the thermal readout are significantly reduced.

## Discussion

In this work we have dealt with the problem of having precise measurements with rare-earth-based thermometers that are typically characterized by a reduced thermal sensitivity. Via the acquisition of multiple luminescence spectra of the selected RENPs at given temperatures, we were able to account for the experimental error in the determination of several thermometric parameters. When applying dimensionality reduction methods, new thermometric parameters were found, and an improvement of the thermal resolution was observed. Specifically, while the best RENP’s thermometric parameter identified in a classical way (i.e., visually inspecting the spectra) provided a thermal uncertainty of 0.15 °C, the best DR-based parameter lowered this value to 0.09 °C. This difference in value was shown to be factual in a comparison of readouts in an experiment where the temperature was slowly changed in steps of 0.2 °C. The best DR-based parameter provided a less noisy estimation of temperature and was able to detect smaller changes in temperature. A similar scenario was observed with Ag_2_S SNCS, where a 2-fold improvement – from 0.13 to 0.06 °C – of the average precision over the explored temperature range was achieved and verified with a quality check experiment. The reason for such superiority resides in the better ability of DR techniques to discriminate between wavelength ranges that correlate differently with temperature (Section S8, Figs. S11 and S12). The herein obtained values outperform those previously reported in works that applied neural networks to infer temperature^43,44^. The conclusions taken of this proof-of-concept study are envisaged to spur researchers to employ better thermometric parameters than the ones used so far, placing emphasis also on data analysis instead of only on the synthesis of novel materials.

This pursuit for reduced thermal uncertainty is particularly relevant in the biological and biomedical context. Firstly, because the signal-to-noise ratio of the detected light is usually lower, and the quality of the thermal sensing is consequently poorer particularly in vivo. Secondly, because several biological processes are associated with temperature changes in the order of 1 °C and sometimes below 0.1 °C^45^. The latter is the case, e.g., with brain response during seizures. Hence, to properly and accurately monitor these processes (particularly their onset), thermal resolutions smaller than 0.1 °C are desirable.

Concerning the readout accuracy, our work suggests that PCA and t-SNE can have a positive or neutral effect, with the possibility of improvement depending on the goodness and statistical significance of the fit of the thermal dependence of the new variables. The data transformation, which inevitably leads to a combination of intensity- and shaped-based features of the spectra, can also play an important role in this sense. Indeed, previous results suggest that a mix of such features is beneficial for the accuracy of the models^43^. Given a proper pre-processing procedure, the approach proposed in this study can be extended to different luminescent thermometers. In this sense, since the first and second-order derivatives of the spectrum are widely used to reduce dispersion, baseline and/or multiplicative effects^36^, the code that we uploaded to Zenodo^46^ allows the user to freely select if they intend to apply such pre-processing to their input data for the DR routines (see also Fig. S13).

At this point of the discussion, a comment about the use of the relative thermal sensitivity, S_r_, as a figure of merit in DR-based approaches is due. While generally in the literature methods are compared based on this figure of merit, we believe that such comparison could be misleading here because the DR algorithms that we utilized involved transforming the original data into quantities that did not always possess an easily interpretable physical meaning. Case in point, the new thermometric parameters resulting from DR could in principle change their sign as temperature varied, i.e., they could cross the zero boundary (as it was the case of PC1 for Ag_2_S SNCs, Fig. 6e). While this does not constitute a problem for the computation of δT, it does for S_r_. In fact, if n is a parameter whose value is zero at a temperature T_c_, then the definition of S_r_ = |dn/dT | /n implies that its value will be indeterminate at T_c_. At a first glance, having a high relative thermal sensitivity seems a desirable aspect. Yet, it does not necessarily carry the meaning that one intuits: One can have extremely high sensitivity but poor thermal resolution, hence leading to a poor thermal readout^32^. What ultimately matters in a calibration is not necessarily the percentual change in the value of a parameter, but how sensitive the detection system is to the changes induced in this parameter. In Fig. S14, it is verified that the DR-based approaches provide from 2 to 10-fold improvements in the value of S_r_ for RENPs. But, for the reasons explained above, i.e. DR-based parameters are not physical quantities strictly speaking, the comparison between their S_r_ values and the ones provided by the classical approach is misleading. Hence, we believe that δT is the most suitable figure of merit in this context and, really, more in general in luminescence thermometry.

## Materials and methods

### Characterization

The morphology and size of the RENPs were investigated using a transmission electron microscope (TEM, JEM1400 Flash (JEOL) microscope). Dynamic light scattering (DLS) measurements were performed on a Malvern Zetasizer Ultra. For recording the photoluminescence, a 0.2-cm optical path cuvette was filled with a RENP dispersion in either ODE or water. The cuvette was placed in a qpod 2e (Quantum Northwest, Inc.), closed using a teflon cap with a hole. A thermocouple was inserted in the cuvette so that its tip was just above the laser path in the dispersion, hence having the minimum temperature discrepancy between the actual temperature along the optical path and the one measured by the thermocouple. A 790-nm laser was used to excite the RENPs or Ag_2_S SNCs. The parameters and conditions used for the acquisition of the spectra are summarized in Table 1. For recording the NIR emission of RENPs and Ag_2_S SNCs, two 850-nm long-pass filters (Thorlabs FEL850) were placed along the emission path to remove the scattered radiation. The emitted radiation was spectrally sorted by a high-brightness monochromator (Shamrock 163, Andor) equipped with two gratings: i) blaze = 1700 nm, 75 g mm^−1^ for NIR emission and ii) blaze = 500 nm, 300 g mm^−1^ for UC (visible) emission. The spectrally sorted emission was detected with an infrared photomultiplier (Hamamatsu H1033C).

As observed in Figure S15, after stabilization the qpod maintains the temperature within the cuvette stable within ± 0.08 or ± 0.047 °C if we consider the standard deviation or the difference between the minimum and maximum temperature value, respectively. The used thermocouple has a resolution of 0.001 °C. The two thermometer calibrations and quality check experiments (RENPs and Ag_2_S SNCs in the NIR) were performed with static dispersions in deionized water.

### Data pre-processing

Before analyzing the data, a set of pre-processing steps were taken. Firstly, the values of intensity corresponding to the wavelengths in which no significant signal had been observed were manually removed. Specifically, the ranges of 900–940, 1102–1288, 1380–1464 and 1627–1700 nm were ignored for the RENPs and the ranges of 900–1000 and 1400–1600 nm for the Ag_2_S SNCs. The spectra were then mean-centered and stored in a matrix of 1500 rows (number of points in the calibration) and 257 (RENPs) or 312 (Ag_2_s SNCs) columns (number of wavelengths in which the intensity was measured)^36^. The columns of such matrix were then, in turn, SNV (Standard Normal Variate) normalized. A second matrix was created calculating the first order derivative (obtained through a Savitzky-Golay matrix of radius 9 (RENPs) or 30 (Ag_2_S SNCs) and order 2) of the original spectra. It also had its columns SNV normalized. This second matrix was meant to highlight changes in the shape of the spectra^36^. For each sample, their two corresponding matrices were then joined and set as the input (calibration) data in the DR models. For the sake of simplicity, this will hereafter be called M^std^.

### Principal component analysis (PCA)

PCA is a technique that transforms, up to a certain degree of accuracy, an amount of *p* correlated variables into *k* uncorrelated ones (where k < p). These new variables are called principal components and they retain as much as possible of the variance present in the original dataset. Its principles are found in linear algebra and, simply put, it finds the principal axes of the ellipsoid generated by the set of data points. In our context of luminescence thermometry, we applied PCA through the following steps:

(i) As all the columns of the M^std^ are already centered around zero, calculate the covariance matrix through:
  $$Cov_{i,j} = \mathop {\sum }\limits_{q = 1}^{1500} \frac{{M_{i,q}^{std}M_{j,q}^{std}}}{{1500 - 1}}$$
(ii) Calculate the eigenvalues and eigenvectors of the covariance matrix.
(iii) Sort the eigenvectors according to their eigenvalues in the descending order. This organizes them according to the variance of the original data that they can account for.
(iv) With a previously stablished criteria (for instance, accounting for 90% of the variance of the original data), find the minimal number, K, of eigenvectors satisfying it.
(v) Calculate the projection, PC, of the standardized luminescence spectrum measured at temperature T_j_ into the r-th eigenvector through:
  $$PC_r^j = \mathop {\sum }\limits_{q = 1}^{N_1} v_r^qM_{q,j}^{std}$$
  where $v_r^q$ is the q-th coordinate value of the r-th eigenvector.
(vi) After obtaining the values of PC for all 1500 measurements and K eigenvectors, study their dependence with temperature.

If one assumes, therefore, that, in a calibration, the only significant physical property that is changed is the temperature, then it is highly likely that the variations in the first principal components take place due to temperature changes. When compared to the usual approach of manually selecting thermometric parameters, one is then safer from the seed of doubt that if a different input feature had been selected, the performance for thermometry would be better.

### t-distributed stochastic neighbor embedding (t-SNE)

t-SNE is a technique that assumes a model probability distribution and verify how much the measured data point deviates from the expected. This difference is called the Kullback-Leibler divergence and must be minimized. In the context of luminescence thermometry, one can proceed as follows:

Similarities in the original space are given by:

$$\rho _{ij} = Exp\left( { - \frac{{\left| {\left| {x_i - x_j} \right|} \right|^2}}{{{\it{\epsilon }}^2}}} \right)/\mathop {\sum }\limits_{k \ne l} Exp\left( { - \left| {\left| {x_k - x_l} \right|} \right|^2/{\it{\epsilon }}^2} \right)$$

where ε corresponds to a neighborhood radius.

On the other hand, similarities in the lower-dimensional space are given by

$$q_{ij} = \left( {1 + \left| {\left| {y_i - y_j} \right|} \right|^2} \right)^{ - 1}/\mathop {\sum }\limits_{k \ne l} \left( {1 + \left| {\left| {y_k - y_l} \right|} \right|^2} \right)^{ - 1}$$

The lower-dimensional embeddings y_i_ are computed by minimizing the function $\mathop {\sum }\nolimits_{i,j} \rho _{ij}\log \left( {\rho _{ij}/q_{ij}} \right)$. The parameter ε is indirectly connected to what we called perplexity parameter in the main text. The higher the perplexity the smaller the number of clusters.

### Projecting the data onto the reduced space

While for PCA the projection of the experimental dataset onto the reduced space was done through calculations of internal products, for t-SNE the matter was not so straightforward. Non-linear approaches were needed to approximate the function that maps the input data into the newly found space^47^. For such, the “DimensionReduction” function of Wolfram Mathematica has been applied.

## Supplementary information

Supplemental Material
