# Supplementary material for: Less is more: dimensionality reduction as a general strategy for more precise luminescence thermometry
Source: Light Sci Appl. 2022 Jul 27;11:237. doi: 10.1038/s41377-022-00932-3 (PMC9329371; doi:10.1038/s41377-022-00932-3)
Supplement: Supplementary file 1 — Supplemental Material [file 41377_2022_932_MOESM1_ESM.docx]

Supplementary Information for:

**Less is more: dimensionality reduction as a general strategy for more precise luminescence thermometry**

Erving Ximendes^1,2^, Riccardo Marin^1^, Luis Carlos Dias^3^, Daniel Jaque^1,2^

^1^ NanoBIG, Departamento de Fısica de Materiales, Facultad de Ciencias, Universidad Autónoma de Madrid, C/Francisco Tomás y Valiente 7, Madrid 28049, Spain

^2^ NanoBIG, Instituto Ramón y Cajal de Investigación Sanitaria (IRYCIS), Ctra. Colmenar km. 9.100, Madrid 28034, Spain

^E-mail:^ [^erving.ximendes@uam.es^](mailto:erving.ximendes@uam.es)^;^ [^riccardo.marin@uam.es^](mailto:riccardo.marin@uam.es)

**Table of Content**

S1. Rare-earth nanoparticle (RENP) synthesis and transfer to water S2

S1. Additional characterization of the RENPs S4

S2. Comparison of the thermometric performance with classical approach S5

S3. Histogram and Statistics of Calibration data points S6

S4. Principal Component Analysis S7

S4. Second coordinate of t-SNE S9

S5. Investigation of the repeatability S10

S7. Additional data for Ag_2_S semiconductor nanocrystals S11

S8. Identification of wavelength ranges with thermal dependence S12

S9. Flowchart for the selection of variables S14

S10. Relative thermal resolution for different thermometric parameters in RENPs. S15

S11. Temperature stability in the qpod S16

**S1. Rare-earth nanoparticle (RENP) synthesis and transfer to water**

**Chemicals.** Lithium trifluoroacetate (LiTFA, Aldrich, 95%), erbium oxide (Er_2_O_3_, Alfa Aesar, 99.9%), ytterbium oxide (Yb_2_O_3_, Alfa Aesar, 99.9%), neodymium oxide (Nd_2_O_3_, Alfa Aesar, 99.9%), yttrium oxide (Y_2_O_3_, Alfa Aesar, 99.99%), trifluoroacetic acid (TFA, Fluka, >99%), 1-octadecene (ODE, Alfa Aesar, 90%), oleic acid (OA, Alfa Aesar, 90%), oleylamine (OAm, Acros Organics, 80-90%), NOBF_4_ (Merck, 95%), trisodium citrate (Na_3_cit, Acros Organics, 98%). Hexane, ethanol, dimethyl formamide (DMF), chloroform (CLF), and acetone were purchased from Labbox, Aldrich, and Fisher BioReagents. The final nanoparticle dispersions were obtained in Milli-Q water.

**Synthesis of core/shell/shell/shell RENPs.** The synthesis of the RENPs was performed following the method reported by Cheng et al., which entails the preparation of seeds (first nuclei) followed by their stabilization via a “wet-annealing” procedure in the presence of oleic acid to promote ripening-driven size-focusing, and successive growth of shells.^47^

**Table S1.** Conditions for the synthesis of the multi-shell RENPs used in this study.

| **Parameter** | **ODE** | | **OA** | | **OAm** | **Other** | **LiTFA** | **RE(TFA)_3_** | **Injection rate** | **Temp.** | **Time** | |
| --- | --- | --- | --- | --- | --- | --- | --- | --- | --- | --- | --- | --- |
| **Unit** | **mL** | **mL** | | **mL** | | **mL** | **mmol** | **mmol** | **mL min^-1^** | **ºC** | **h** | |
| **Core preparation** | | | | | | | | | | | |  |
| **First nuclei** | 14^a^ + 6 | | 7 ^a^ + 3 | | 7 ^a^ + 3 |  | 2.5 | 2.5 | 1.5 | 330 | 1 | |
| **Stabilization** |  | | 16 | |  | 8 (nuclei) |  |  |  | 320 | 1 | |
|  |  | |  | |  |  |  |  |  |  |  | |
| **Shelling** | | | | | | | | | | | |  |
| **Core mixture** | 7.5 | | 7.5 | |  | 4.8 (cores) |  |  |  |  |  | |
| **Shell 1** | 5 | | 5 | |  |  | 0.2 | 0.2 | 1.0 | 315 | 0.5 | |
| **Shell 2** | 5 | | 5 | |  |  | 0.2 | 0.2 | 1.0 | 315 | 0.5 | |
| **Shell 3** | 10 | | 10 | |  |  | 0.4 | 0.4 | 1.5 | 315 | 0.5 | |

**Table S2.** Composition of each RENP volume and quantities used for the preparation of the corresponding RE(TFA)_3_ precursors.

|  | **Y^3+^** | **Y_2_O_3_** | **Yb^3+^** | **Yb_2_O_3_** | **Er^3+^** | **Er_2_O_3_** | | **Nd^3+^** | | **Nd_2_O_3_** | |
| --- | --- | --- | --- | --- | --- | --- | --- | --- | --- | --- | --- |
|  | **% [mmol]** | **mg [mmol]** | **% [mmol]** | **mg [mmol]** | **% [mmol]** | | **mg [mmol]** | **% [mmol]** | **mg [mmol]** | |  |
| **Core** | - | - | - | - | 100 [2.5] | 478.2 [1..25] | | - | | - | |
| **Shell 1** | 85 [0.17] |  | 15 [0.03] | 5.9 [0.015] | - | - | | - | | - | |
| **Shell 2** | 85 [0.17] |  | 10 [0.02] | 3.9 [0.01] | - | - | | 5 [0.01] | | 1.7 [0.05] | |
| **Shell 3** | 100 [0.4] | 45.2 [0.2] | - | - | - | - | | - | | - | |

**Synthesis of LiErF_4_ first nuclei.** 1.25 mmol (478.2 mg) of Er_2_O_3_ were added to a 50-mL three-neck round-bottom flask along with a stirring bar, 5 mL of TFA and 5 ml of H_2_O and stirred at 80 ºC until complete dissolution of the solid. Subsequently the stirring was stopped, the temperature lowered to 60 ºC, and the obtained Er(TFA)_3_ precursor was left to dry overnight. The next day, 14 mL ODE, 7 mL OA, and 7 mL OAm were added to a 50-mL three-neck round-bottom flask along with a stirring bar. The mixture was degassed at 125 ºC for 30 min and later the temperature was increased to 330 ºC under a protective Ar atmosphere. In the meantime, the precursor solution was prepared by adding 6 mL ODE, 3 mL OA, 3 mL OAM, and 2.5 mmol (300 mg) LiTFA to the dry Er(TFA)_3_, and degassing at 125 ºC for 30 min (all the solid was dissolved at that time point). The precursor solution was subsequently loaded in a 20 mL glass syringe and injected into the first mixture at 330 ºC with the aid of an automated injector (New Era Pump System Inc., model # NE-300) at a constant rate of 1.5 mL min^-1^. Once the injection was terminated, the reaction was allowed to proceed for 1 h. After this time, the heating was stopped and the mixture allowed to cool down to room temperature naturally. The crude reaction product was stored as is in a 50-mL centrifuge tube. The concentration of this dispersion was estimated at 0.0625 M of RE^3+^ (considering 100% chemical yield). A 1.5-mL aliquot of the first nuclei was washed adding excess ethanol, followed by centrifugation at 3500 *g* for 20 min. The supernatant (containing a small fraction of well separated, viscous yellow fraction) was discarded, and the pellet resuspended in 0.5 mL hexanes. Precipitation with ethanol, centrifugation, and resuspension were repeated three times and finally the particles were resuspended in 1 mL hexanes for further characterization.

**Stabilization of LiErF_4_ core.** 8 mL of the first nuclei dispersion was added to 16 mL of OA. The dispersion was degassed at 110 ºC for 30 min and later heated to 320 ºC under Ar atmosphere, allowing the annealing to proceed for 1 h. After this time, the reaction was stopped, and the mixture allowed to cool down at room temperature naturally. The stabilized cores were stored as is in a 50-mL centrifuge tube. The concentration of this dispersion was estimated at 0.021 M of RE^3+^. A 1.5-mL fraction was washed as described above and stored in hexanes for further characterization.

**Successive shell growth.** To grow the multi-shell RENPs targeted (see Table S1 for the composition of each shell, the quantities of precursors, volumes of solvents, and injection rates used for each step), 4.8 mL of the core RENPs were added to a 100-mL three-neck round-bottom flask along with a stirring bar and 7.5 mL OA and 7.5 mL ODE. The dispersion was degassed at 110 ºC for 30 min and later heated to 315 ºC under a protective Ar atmosphere. In the meantime, the shelling solution was prepared mixing solvents and precursors listed in Table S1 and degassing them at 110 ºC for 30 min. The shelling solution was subsequently injected into the solvent mixture at 315 ºC at the rate specified in Table S1, and the reaction allowed to proceed for 30 min. Just before injecting the next shelling solution, a 1.5-mL aliquot was sampled and washed according to the procedure described above. After the growth of the third shell, the RENPs were allowed to cool to room temperature naturally, washed as described above, and dispersed in 5 mL hexanes for further characterization. For the study of the luminescence thermometry, 1 mL of the sample was diluted in 1 mL ODE, along with one drop of OA. The mixture was heated at 70 ºC and left under stirring at that temperature for 30 min. The resulting RENP dispersion in ODE was stored at 8 ºC as a solid and tawed as needed for the measurements.

**Transfer to water.** The RENPs were transferred to water using a ligand exchange procedure based on the method reported by Dong et al.^48^ Briefly, 800 μL of RENPs in hexanes was precipitate by addition of excess ethanol and centrifuged at 6000 *g* for 10 min. The RENPs were redispersed in DMF by sonicating to yield a turbid dispersion. This dispersion was added to a glass vial along with a stirring bar and stirred at 1000 rpm. 5 mg of NOBF_4_ were added to the vial, followed by 5 min of stirring, 5 min of sonication, and an additional 10 min of stirring. Subsequently, 3 mL of chloroform were added to precipitate the RENPs and dissolve the removed OA. The mixture was centrifuges at 6000 *g* for 5 min, the supernatant discarded, and the RENPs finally redispersed in 1 mL DMF in a glass vial. To this dispersion, 3 mL of a 0.5 M solution of Na_3_cit in water was added dropwise, followed by 20 min of stirring at 1000 rpm, and 10 min of sonication. The dispersion was split in two 15-mL centrifuge tube, and 8 mL acetone was added to each of them. The RENPs were recovered by means of centrifugation at 6000 *g* for 20 min and redispersed in distilled water. The washing procedure (precipitation with 4x excess acetone, centrifugation, and redispersion in water) was repeated three times, and the RENPs were finally redispersed in 1 mL of distilled water and stored at 8 ºC for further characterization.

**Additional characterization of the rare-earth nanoparticles (RENPs)**


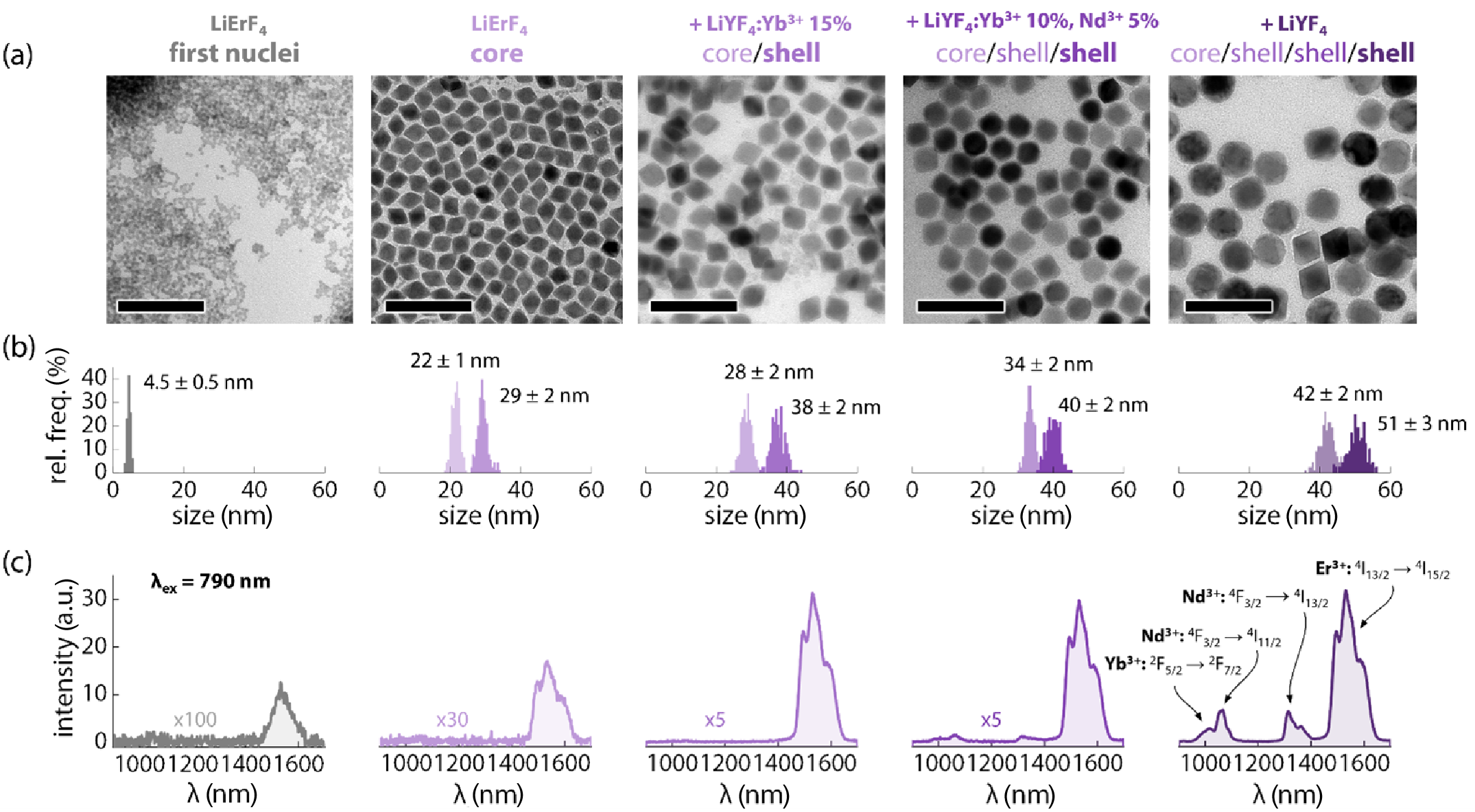


**Figure S1.** **Characterization of multishell rare-earth nanoparticles (RENPs).** a) Transmission electron microscope (TEM) images of the RENPs at different growth stages from the stage of first nuclei to the triply shelled RENPs with composition LiErF_4_/LiYF_4_:Yb^3+^ 15%/ LiYF_4_:Yb^3+^ 10%, Nd^3+^ 5%/ LiYF_4_. Scale bars are 100 nm. b) Size distributions obtained from the analysis of 200 RENPs. While the first nuclei have a quasi-spherical morphology, at the other growth stages the RENPs acquire a bipyramidal habitus typical of many LiREF_4_ structures, with a short and a long axis. The two histograms reported for each RENP stage are thus respectively for the short and long axis. c) Near-infrared emission spectra of the RENPs at different growth stages obtained under 790-nm excitation, with a power density of approximately 3.0 W·cm^-2^ and roughly keeping the RENP concentration (in number) constant. All but the spectrum of the final core/shell/shell/shell RENPs were multiplied by a variable factor (indicated in the graphs) for the sake of clarity. The appearance of different signals arising from the subsequently added rare-earth ions, the trend of the emission intensity, along with the steady size increase of the RENPs together confirm the success of the shelling procedure. The final inert shell of LiYF_4_ was grown to increase the overall emission intensity of the RENPs, thanks to the passivation of surface states/defects and shielding of the luminescent inner rare-earth ions from solvent molecules.


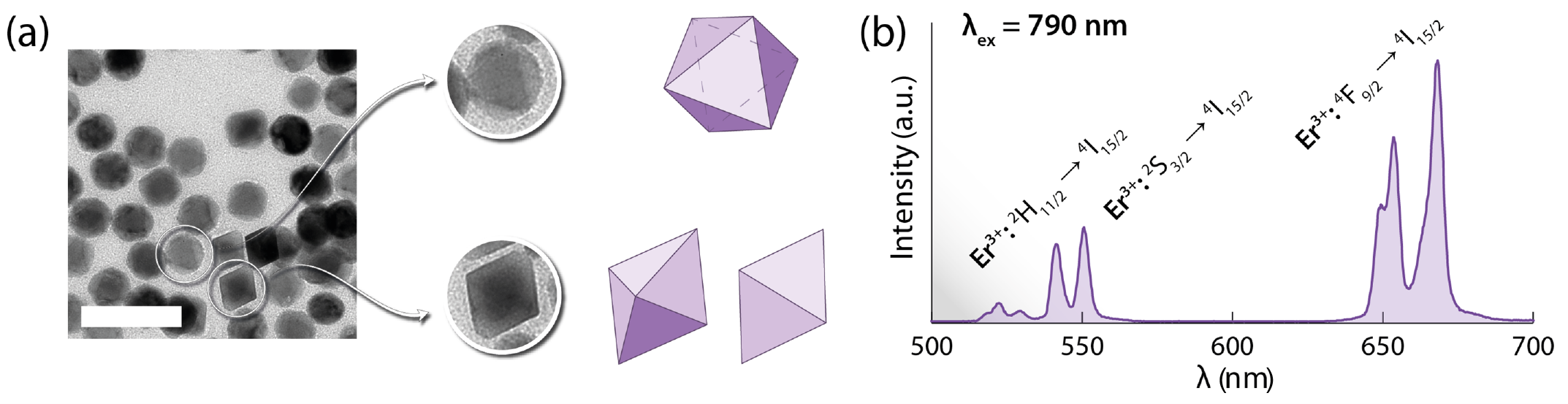


**Figure S2.** a) Zoom in of two nanoparticles with different spatial orientation along with sketches of their 2D appearance during TEM observation. Scale bar is 100 nm. b) Upconversion emission spectrum of RENPs under 790-nm excitation.

**S2. Comparison of the thermometric performance with classical approach**

**Table S3.** Comparison of the relative thermal sensitivity between the proposed RENPs and other RENPs working in the NIR. Only colloidal nanoparticles measured in aqueous dispersions are herein reported.

| **Host material** | **Max S_r_ (%·ºC)** | **T of max S_r_ (ºC)** | **Work** |
| --- | --- | --- | --- |
| NaGdF_4_:Yb^3+^,Er^3+^,Ho^3+^/ NaGdF_4_:Yb^3+^/ NaGdF_4_:Yb^3+^,Nd^3+^/NaGdF_4_ | 1.1 | 20 | 10.1039/C6NR08472A |
| LiErF_4_:Ce^3+^/LiYF_4_ | 0.45 | 20 | 10.1002/adom.202001178 |
| LiLuF_4_:Nd^3+^/LiLuF_4_ | 0.58 | 20 | 10.1039/C9NR02801C |
| NaYF_4_:Yb^3+^, Nd^3+^ | 1 (lifetime-based) | 25-45 | 10.1038/s41598-019-49291-x |
| NaYF_4_/NaYF_4_:Nd^3+^,Yb^3+^/CaF_2_ | 1.5 (lifetime-based) | 60 | 10.1016/j.physb.2021.413652 |
| Y_2_O_3_:Yb^3+^,Er^3+^,Ho^3+^ | 1 | 25-65 | 10.1002/adom.201901173 |
| LiErF_4_/LiYF_4_:Yb^3+^/LiYF_4_:Yb^3+^,Nd^3+^/LiYF_4_ | 1.1 | 31-45 | This work |

**S3. Histogram and Statistics of Calibration data points**

*Traditional thermometric parameters*

To better represent the distribution of values assumed by I_1550_, *R_1_* and *R_2_* during the calibration, a histogram (100 bins with width of 15) of their 1,500 measurements was plotted for each of these parameters. The results are included in **Figure S3** and, as we can see, *R_1_* was the one which presented the least level of overlap between the measurements under the 15 different temperatures. Notice that it had 15 well-identifiable peaks with their distributions reasonably well-separated from each other. The other two parameters, on the other hand, did not satisfy these conditions. Thus, justifying the selection of *R_1_* as the most appropriate, among the traditional parameters, for building a regression model for the temperature.


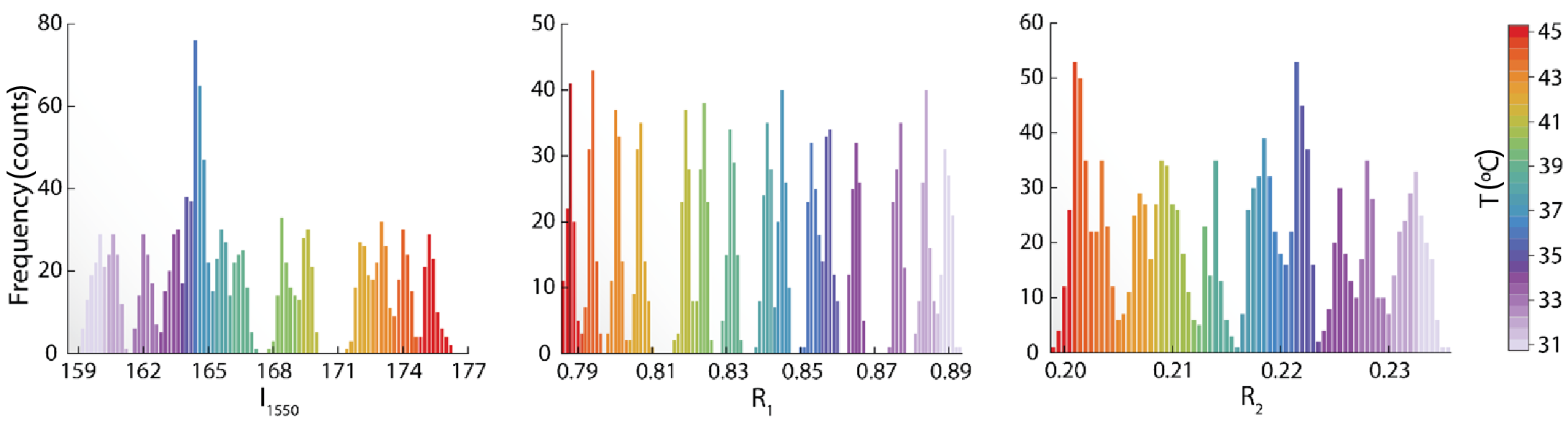


**Figure S3.** **Histogram of common thermometric parameters.** Histogram of the values assumed by I_1550_ (left), R_1_ (middle) and R_2_ (right) in the calibration experiment described in the main text. The graphs were color-coded according to the palette on the rightmost part of the figure.

*DR-based parameters*

The same study was performed on the two DR-based parameters: *PC1* and *t-SNE1*. The results are included in **Figure S4** and they show that both of them have low levels of overlap between the measurements for the 15 different temperatures of the calibration. While both had 15 peaks, they were more easily identifiable, and their distributions were more separated with *PC1* rather than with *t-SNE1*. This observation underlines that *PC1* is the most appropriate, among the DR-based parameters, for building a regression model for temperature.


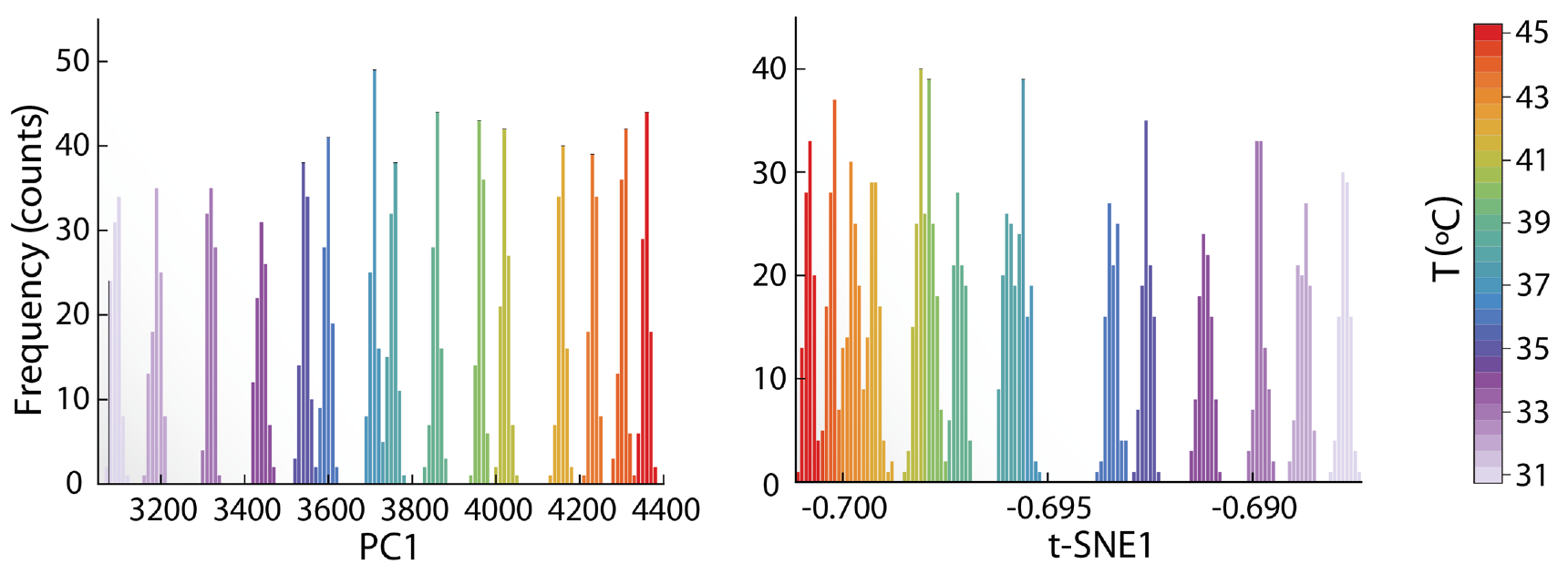


**Figure S4.** **Histogram of DR-based thermometric parameters.** Histogram of the values assumed by PC1 (left), and t-SNE1 (right) in the calibration experiment described in the main text. The graphs were color-coded according to the palette on the rightmost part of the figure.

**S4. Principal Component Analysis**

*Eigenvalues and variance*

After inserting the matrix *M^std^* described in the Methods Section as the input data for PCA, the eigenvalues included in **Figure S5** were the ones found for its covariance matrix. Their sum corresponds to 100% of the variance of the data. Note that considering only the intensity variations, the percentage of variance accounted for by the first eigenvalue (*PC1*) is approximately 83%. However, when considering also the shape of the spectrum (i.e., 1^st^ derivative), to account for 90%, one would need 22 eigenvectors, with *PC1* alone only accounting for approximately 63% of the variance.


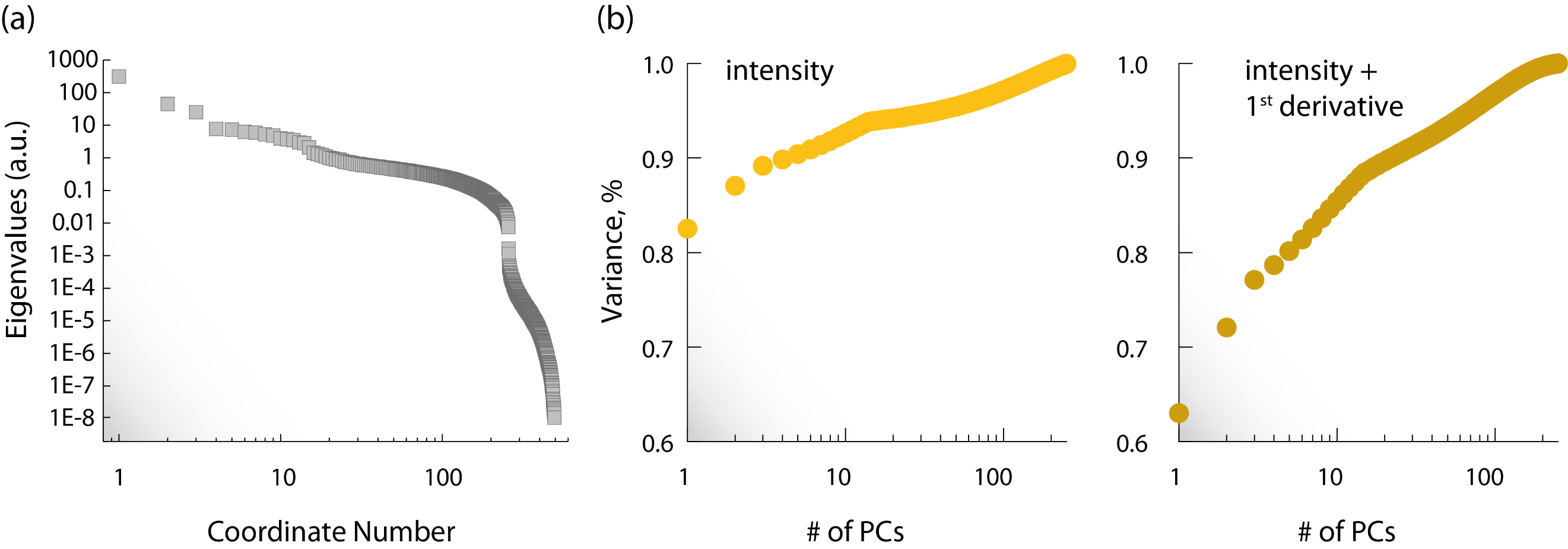


**Figure S5. Eigenvalues and variance.** a) Eigenvalues found for the covariance matrix of the calibration data. b) Variance explained when taking into account the first N eigenvectors either considering only the intensity (left) or intensity and spectral shape/shift (i.e., the first derivative – right).

*Principal Components and their dependence with Temperature*

For the sake of a better description, we include in **Figure S6** the coordinates of the first three eigenvectors in the original space of variables. On the bottom of the Figure, however, we include the dependence with temperature of the projection of the calibration onto these eigenvectors. As one can observe, *PC1* is the only one characterized by an inversible function.


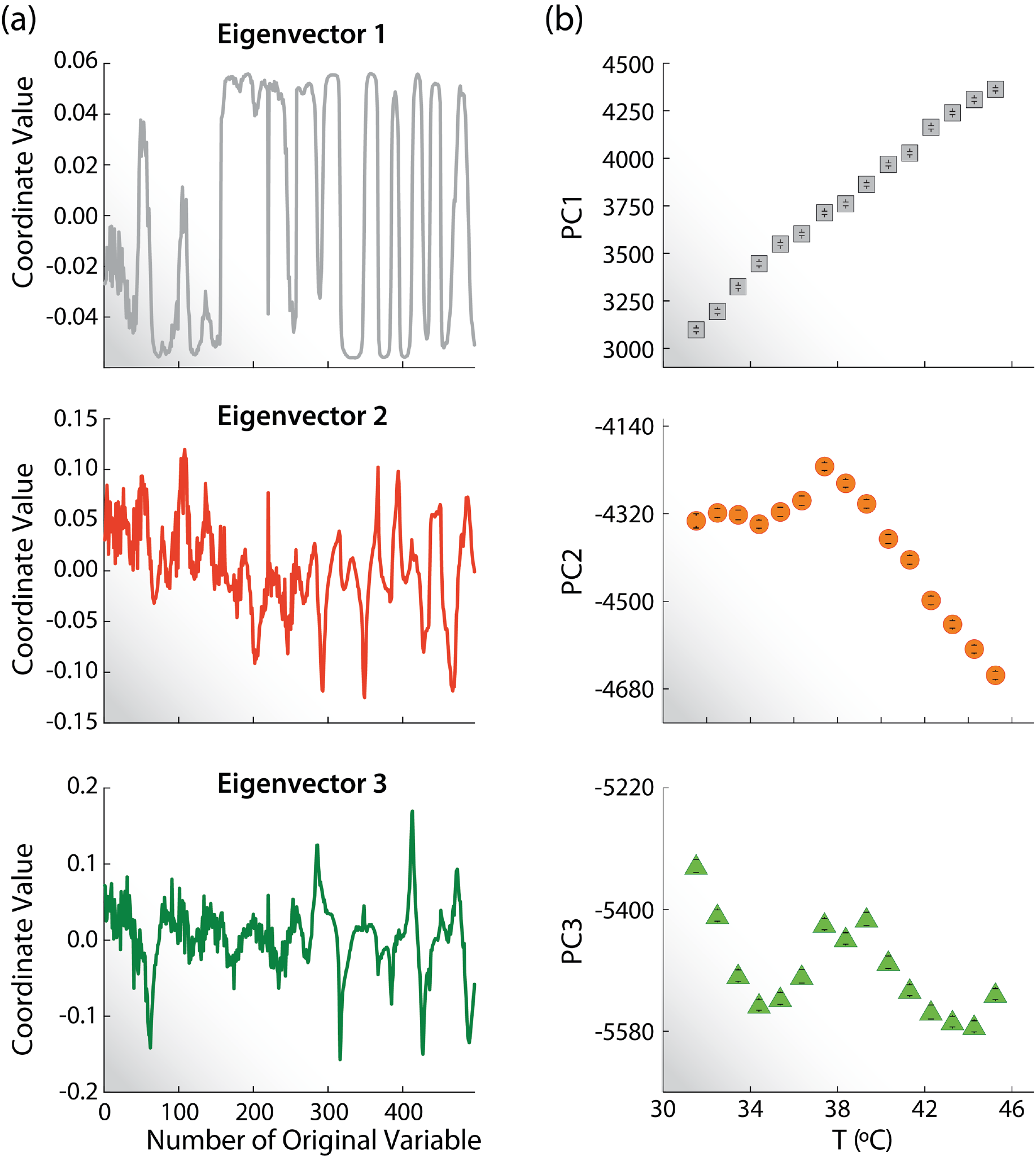


**Figure S6. PCs and their thermal dependence.** a) The coordinates of the first three eigenvectors in the original space of variables. b) Thermal dependence of the first three principal components.

**S5. Second coordinate of t-SNE**


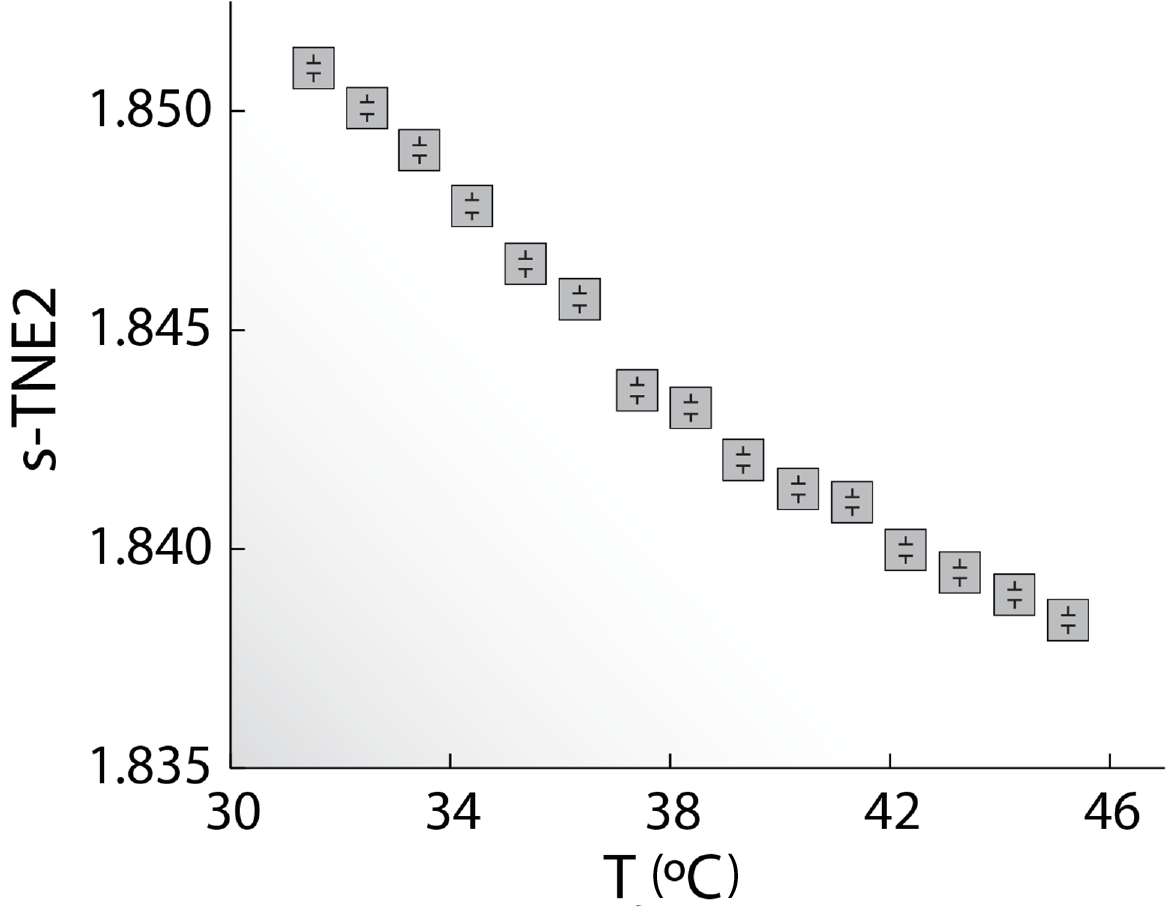


**Figure S7.** Dependence of the second coordinate of t-SNE with the temperature.

**S6. Investigation of the repeatability**

To demonstrate this, we have performed the widely accepted test for repeatability, i.e., performing heating and cooling cycles from a given temperature to another and checking how the thermometric parameters repeat their values over these changes. In our case, a total of 10 cycles were performed. **Figure S9a-e** presents the main results that we obtained. As one can see, apart from *I_1550nm_* and *R_2_*, the parameters seem to have similar behaviour, presenting almost no change when returning to a given temperature. To account for that and make a comparison, we use the definition of Ref 15 for the repeatability, $\rho$, as calculated from a parameter $\Delta$:

$$\rho=1-\max\left( \Delta_{c,i}-\Delta_{i}^{j} \right)/\Delta_{c,i}$$

$$\forall j\in\{1,2,3,4,5\}$$

where $\Delta_{c,i}$ stands for the expected value of $\Delta$ at a temperature *T_i_* according to the fit of the calibration curve and $\Delta_{i}^{j}$ to one of the possible 5 experimental values of $\Delta$ at a temperature *T_i_*. The results are included in **Figure S9f** and they reflect that DR-based approaches do not provide any noticeable improvement in the value of $\rho$. As a matter of fact, R2, PC1 and t-SNE1 presented values of 99.6, 99.4 and 99.9 %. While at a first glance this might be surprising, when retrieving the meaning of *r*, one can notice why it is not. It is worth noticing that the repeatability, *r*, is an estimation of the chance that hysteresis is not playing a role in your thermometer. Thus, if you carefully avoid the conditions that could cause hysteresis, you will increase the value of *r*. In our case, we had avoided both precipitation and degradation of the NPs in such a way that there was almost no room for improvement in the value of $\rho$ (as demonstrated by the value provided by *R_2_* which is already quite close to 1). Thus, any changes that one could see in $\rho$ could only take place in the third decimal place.


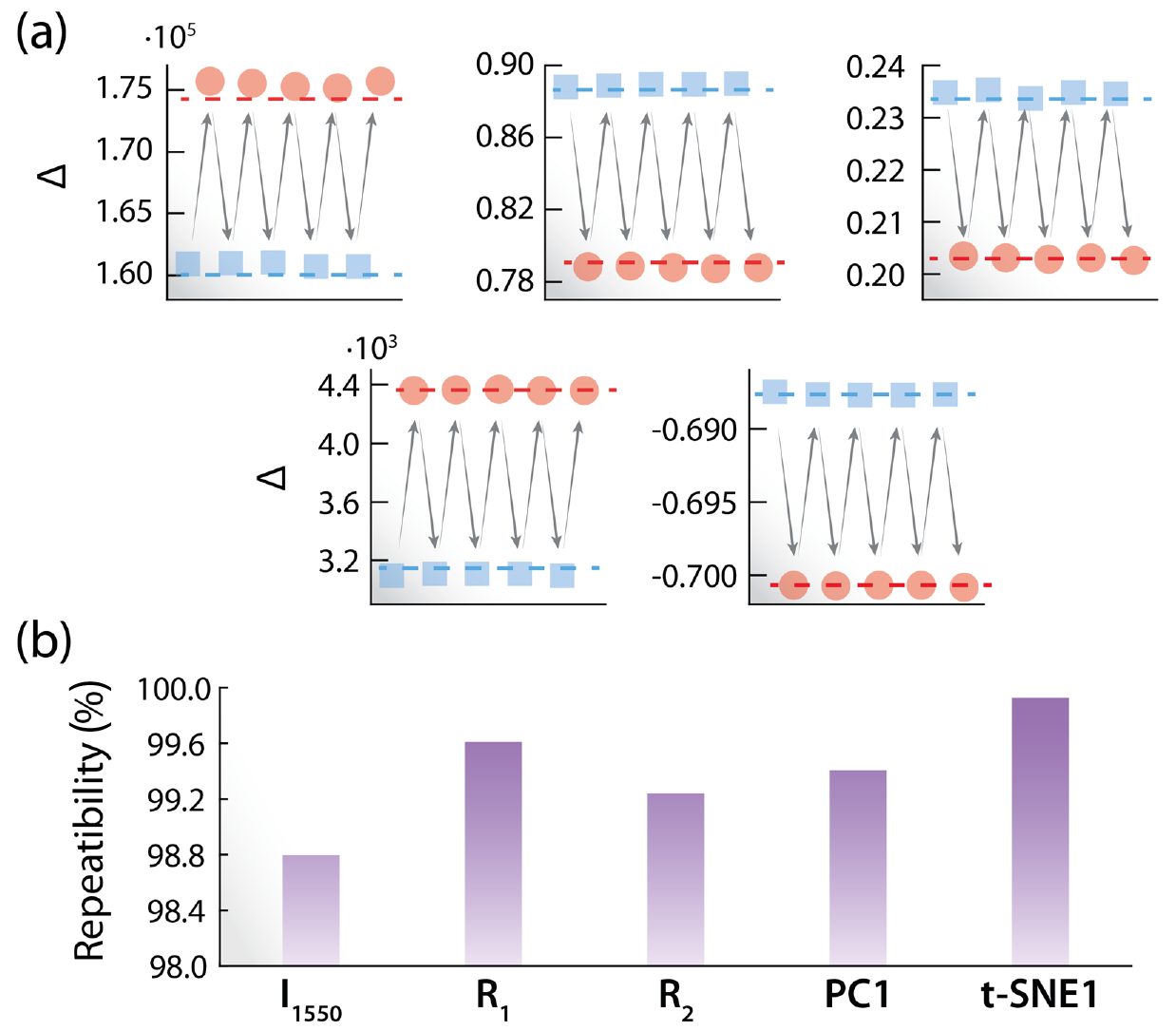


**Figure S8. Repeatability studies.** **a)** Values of *I_1550_, R_1_, R_2_, PC1, t-SNE1* under heating and cooling cycles from 32 (blue squares) to 45 ºC (red circles). The horizontal dashed lines correspond to the expected value of the parameters, according to the fit of the calibration curve, at these two temperatures. **b)** Repeatability of the luminescent thermometer as calculated from its possible thermometric parameters.

That being said, we need to recognize that having control over the circumstances on which hysteresis could occur is a pre-requisite for applying DR-based approaches. And that is so because DR techniques look for the variables that most explain the variance contained in the dataset. And the better the calibration, the more this variance is correlated with temperature.

**S7. Additional data for Ag_2_S semiconductor nanocrystals.**


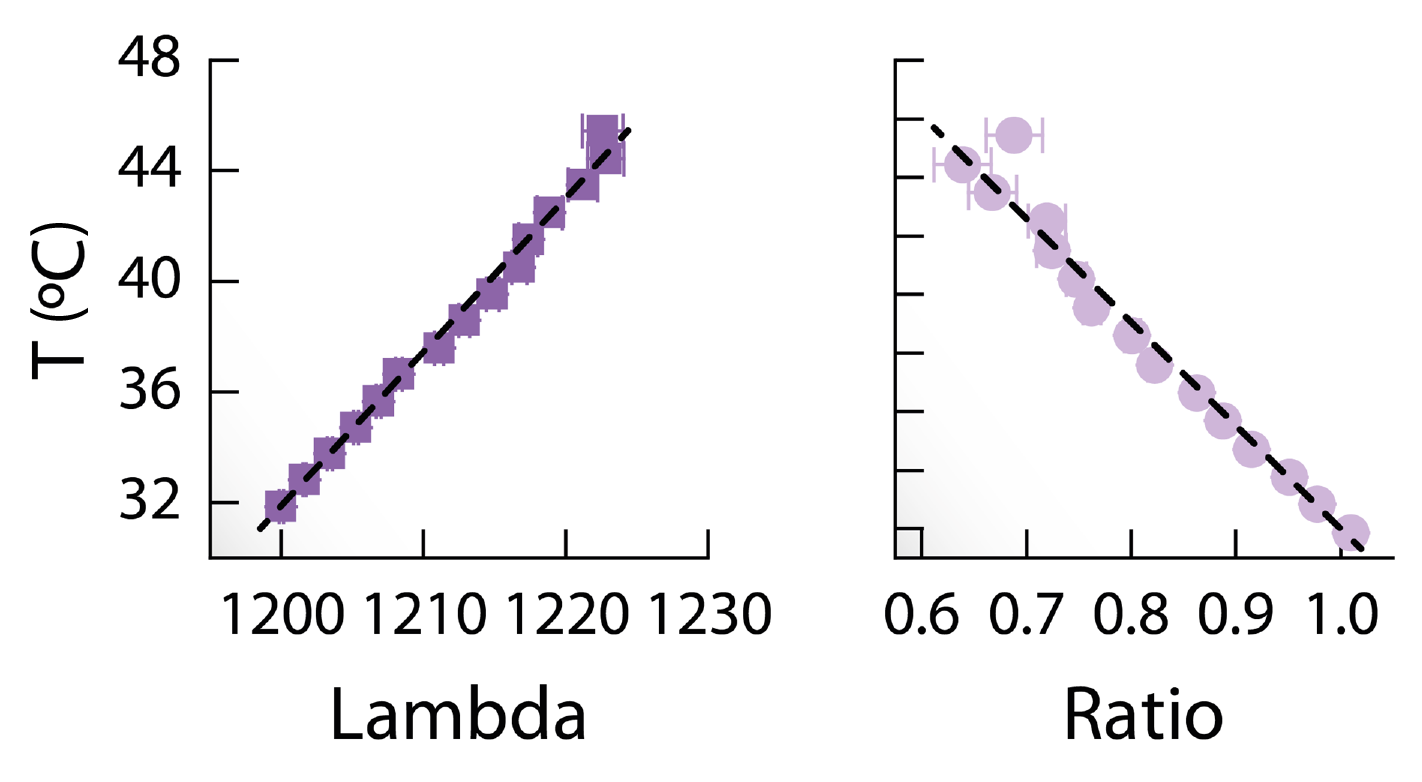


**Figure S9.** Calibration curves using two additional traditional thermometric parameters: the position of the emission peak maximum (lambda) and the intensity ratio (measured considering the integrated intensity over the two wavelength ranges 1000-1200 nm and 1200-1400 nm). Both parameters have a linear relationship with temperature and, as such, were also used to build the MLR model.


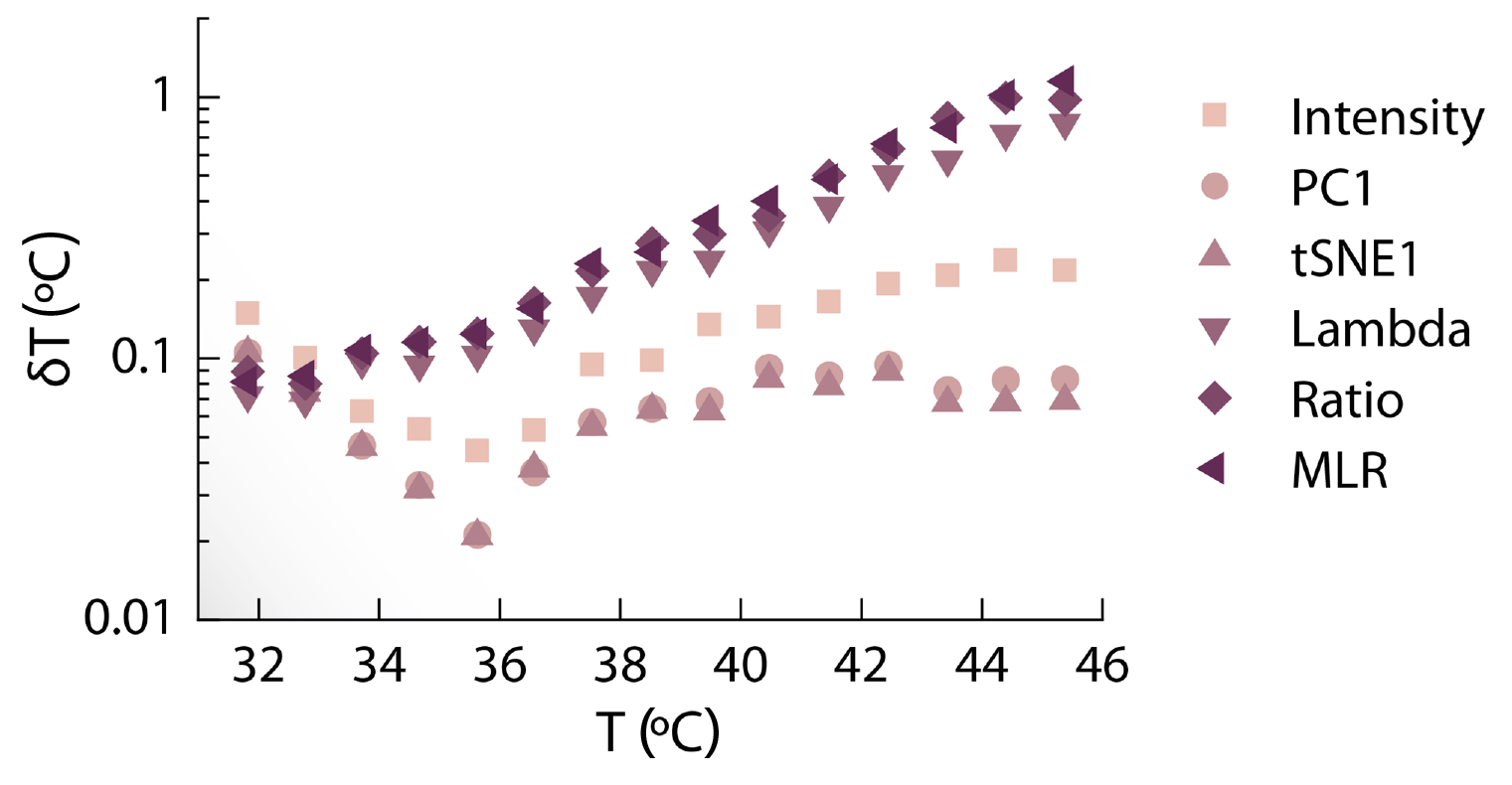


**Figure S10.** Comparison of the thermal resolution for different thermometric parameters for Ag_2_S SNCs .**S8. Identification of wavelength ranges with thermal dependence**

One of the advantages of dimensionality reduction techniques resides in the ability to easily discern wavelength ranges with the most pronounced thermal dependence and whose use in luminescence thermometry maximizes the performance of the thermometric approach.

To that end, we closely analyse here how Principal Component Analysis (PCA) compares with the more classical approach of “educated guess” that is generally applied in luminescence thermometry. Specifically, close inspection of the first PCA eigenvector of the covariance matrix indicates that that the three emission ranges have similar importance in explaining the dataset (with a certain preference given, of course, to the 1550 nm emission band – **Figure S11a**). What is extremely relevant, however, is noticing that PCA finds how these ranges either agree or disagree between themselves with changes in temperature. **Figure S11a**, for instance, indicates that the general behaviour of the 1330 nm emission band is completely opposite of the one centred at 1550 nm (their scores have different signs). It is this alternating change of sign in the scores that allows to get better thermometric parameters. When checking the spectra at different temperatures (**Figure S12a** and **3b** in the main manuscript), one could certainly guess some thermometric parameters. Indeed, that was what was done in the section describing the common approach to luminescence thermometry (i.e., we looked for changes in the emission intensity, intensity ratio, and/or shape of the spectrum). While this modus operandi is reasonable enough, it cannot place enough emphasis on wavelengths ranges with different behaviour from their immediate neighbours.

This is clear observing the emission band corresponding to the Er^3+^: ^4^I_13/2_ → ^4^I_15/2_ transition around 1550 nm. The common approach would simply tell us to integrate the whole emission intensity over the 1420-1670 nm range and study its dependence with temperature. Alternatively, a tedious trial-and-error procedure should be followed to identify the integration intervals that maximize the thermometric performance. When applying PCA (**Figure S11a**), however, we notice a seemingly odd feature: there is a wider range (1460-1605 nm) that has positive scores and a narrower one (1605-1630 nm) that has negative scores. This means that, when the temperature changes, these emission ranges have a negative correlation. To demonstrate that this is the case, the values I_1460-1605_ and I_1605-1630_ of the integrated emission over these ranges are included in **Figure S12b**. While I_1460-1605_ increases with temperature, I_1605-1630_ decreases (though in an exponential way).

A similar case can be made for the 1000 and 1330 nm emission bands. In the latter, there is an even more interesting pattern: PCA detects three ranges, two with negative scores (940-1015 nm and 1035-1105 nm) and one with positive ones (1015-1035 nm – **Figure S11a**). The sum of the integrated intensity over the first two ranges is plotted together with the integrated intensity over the last one in **Figure S12c**. Once again, we notice how PCA could verify different patterns even in short wavelength intervals. Had we followed the common approach, we would add contributions with opposite correlations with temperature.


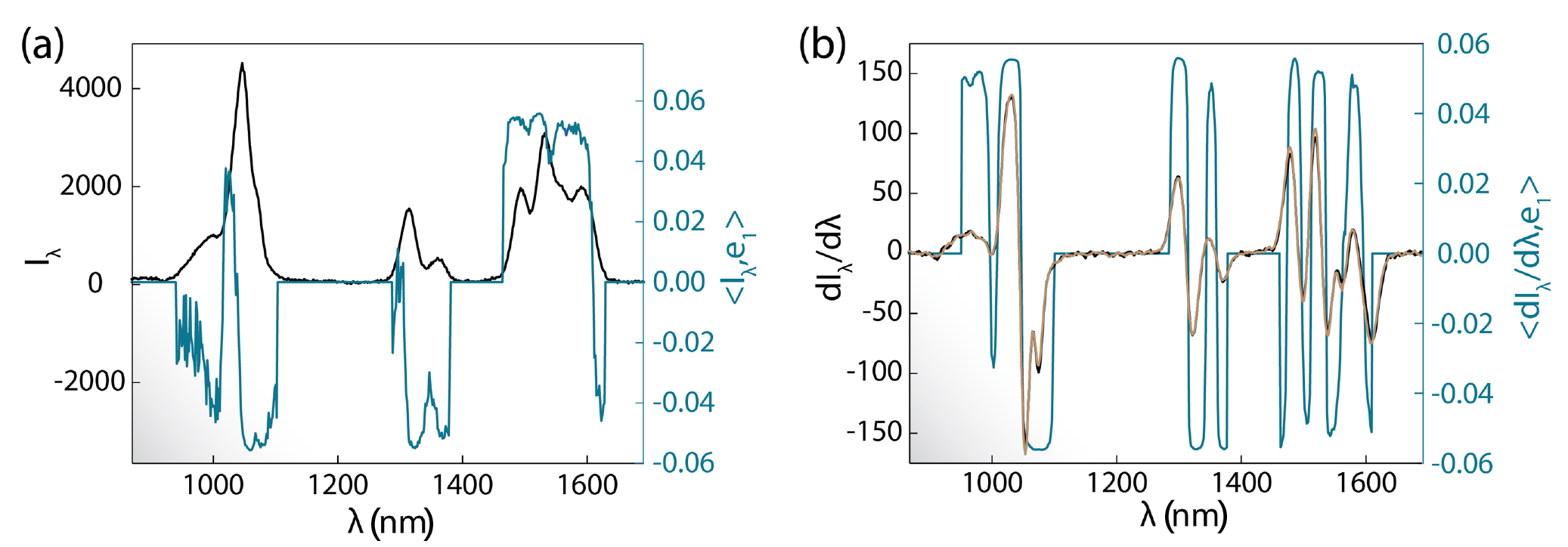


**Figure S11. Importance of spectral ranges in Principal Component Analysis.** Coordinates of the first eigenvector (blue lines) superimposed to a) the luminescence intensities and b) first-order derivative of the intensity to which they correspond. Black and brown curves in (b) correspond to 32 and 46 ºC, respectively.


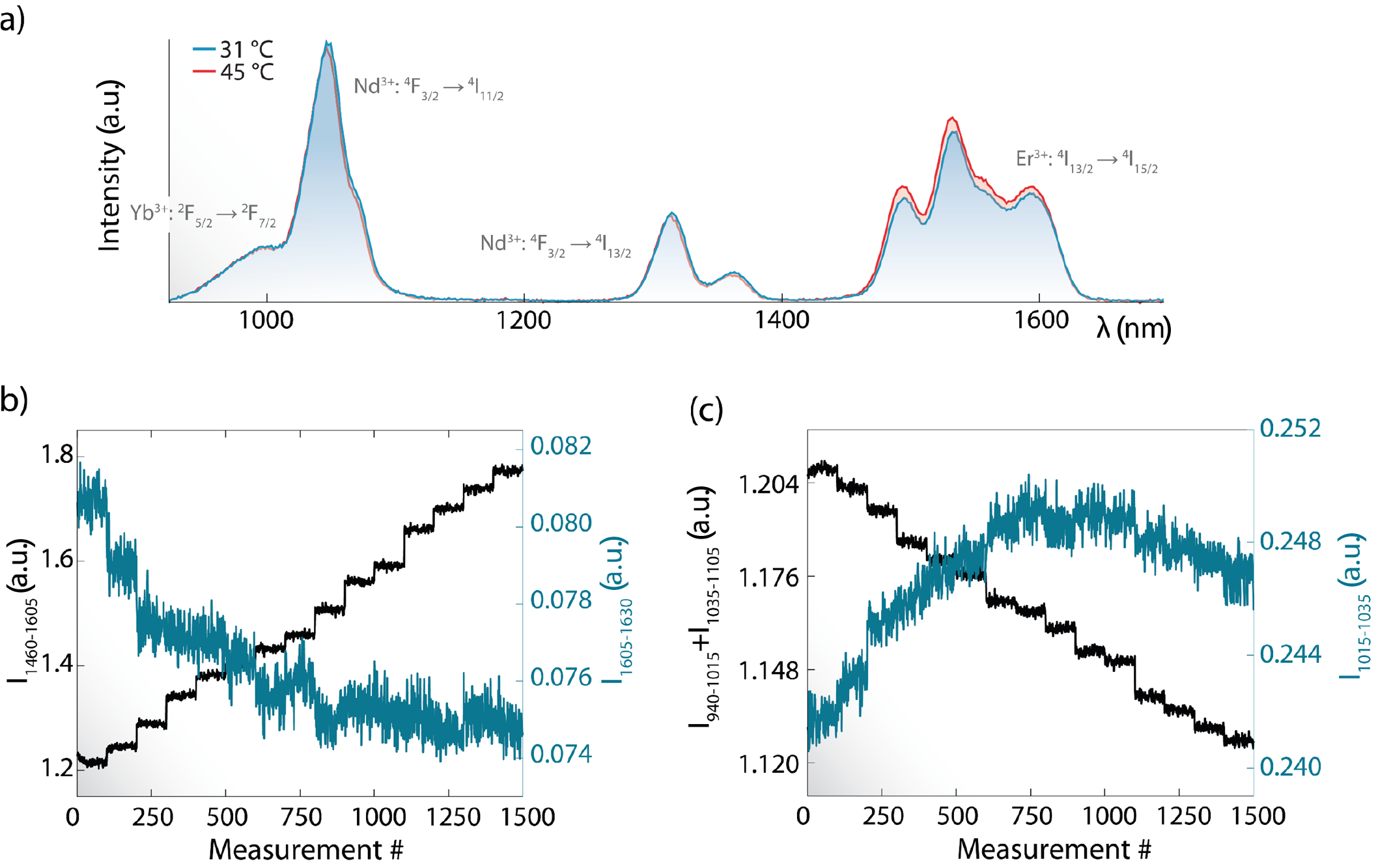


**Figure S12**. **a)** Luminescence spectra of the RENPs used in the work measured at different temperatures. **b)** I_1460-1605_ and I_1605-1630_ as measured throughout the calibration. c) I_940-1015_ + I_1035-1105_ and I_1015-1035_ as measured throughout the calibration.

When it comes to the shape of the spectrum, **Figure S12b** seems to indicate that the guesswork in common approaches could have worked reasonably well, since the PC scores have very similar absolute values (i.e., equal importance to all the emission ranges), and their signs agree with the signs assumed by the derivative. It is important to point out, however, that this might have been the case due to the almost negligible redshift observed during the calibration. Had it been more pronounced in the 31-45 ºC range, the analyses could yield different results.

**S9. Flowchart for the selection of variables**


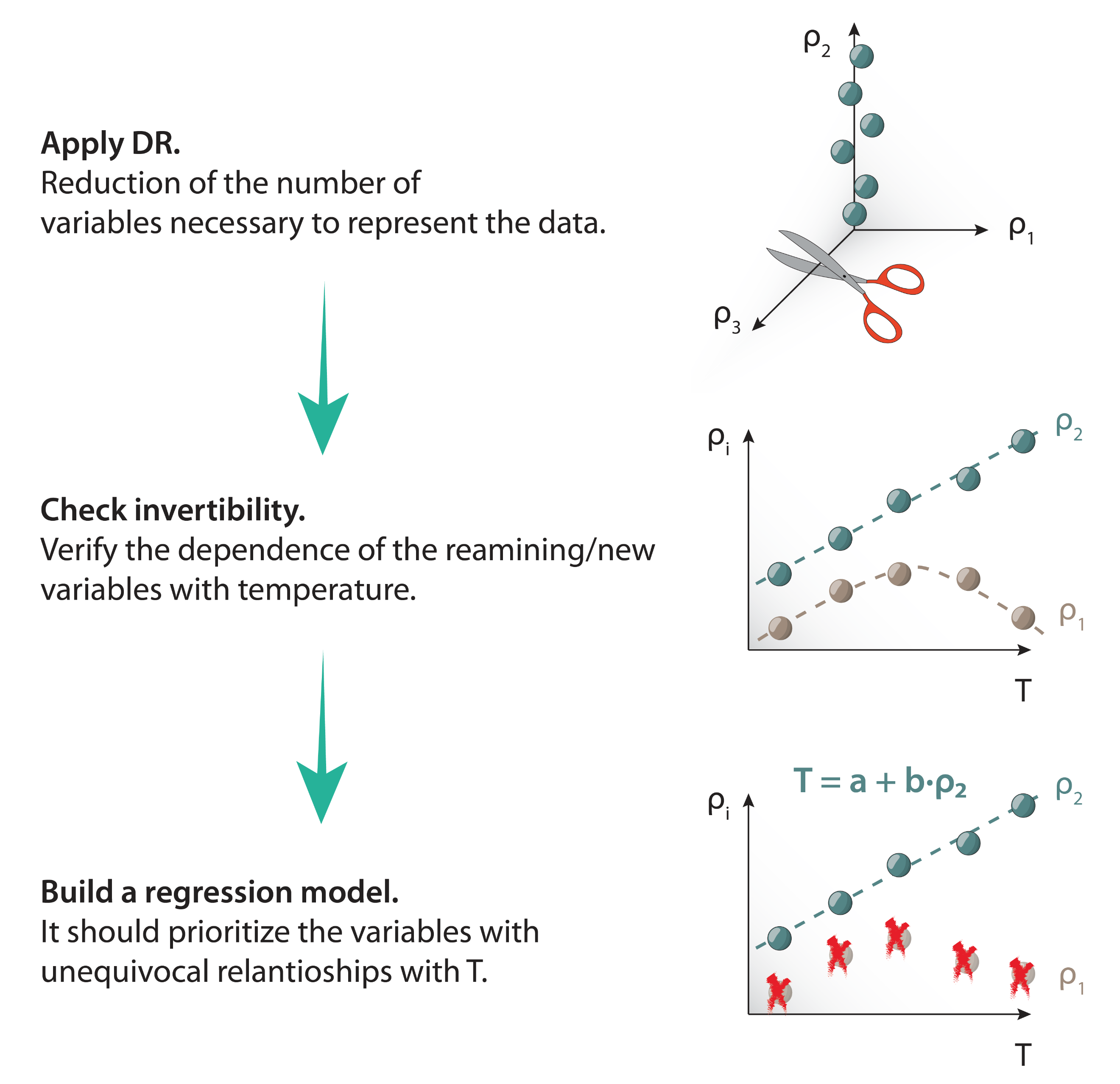


**Figure S13.** Summarized flowchart for the selection of DR variables.

**S10. Relative thermal resolution for different thermometric parameters in RENPs.**


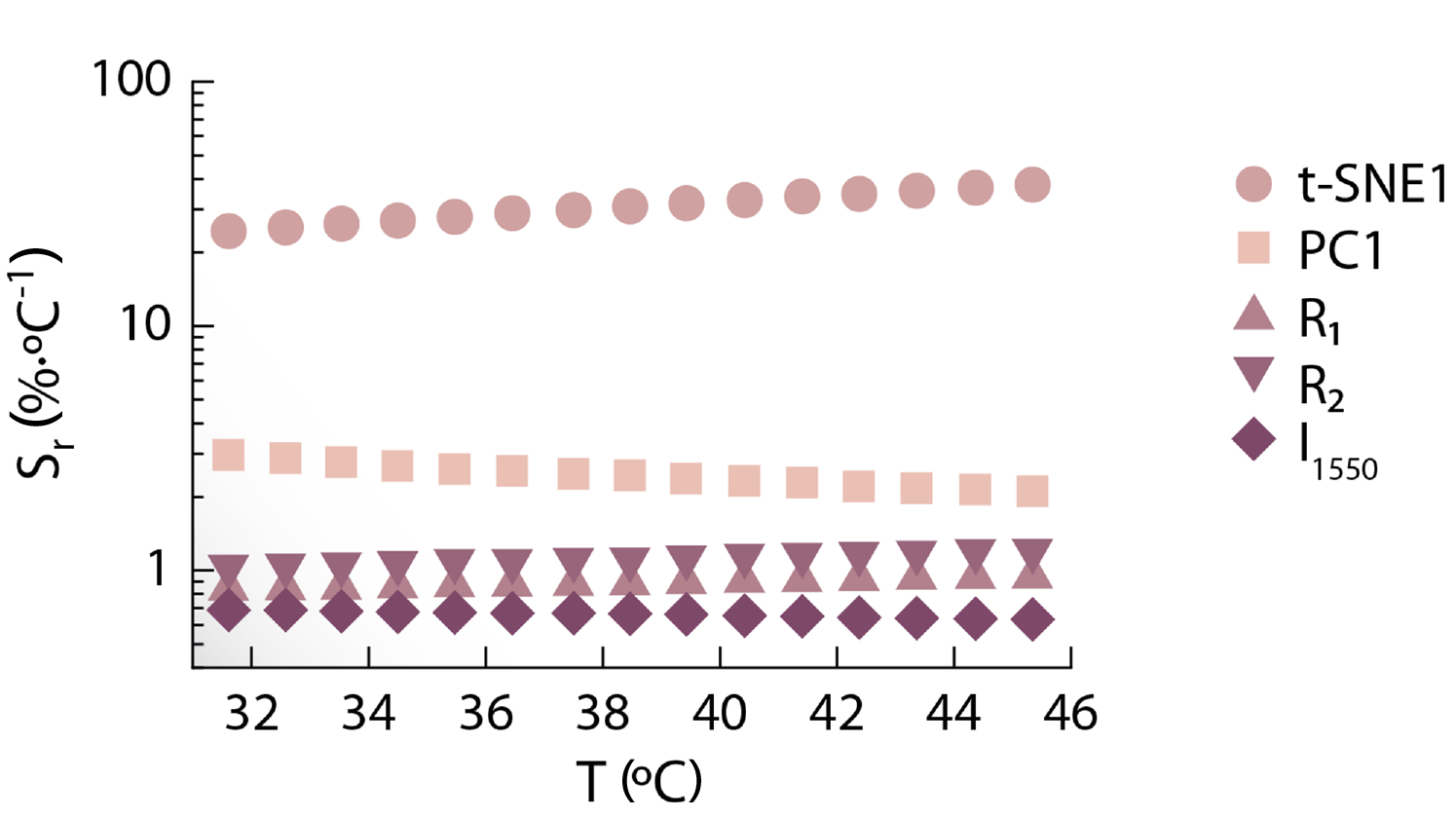


**Figure S14.** Comparison of the relative thermal resolution calculated for different classical and DR-extracted thermometric parameters for RENPs.

**S11. Temperature stability in the qpod**


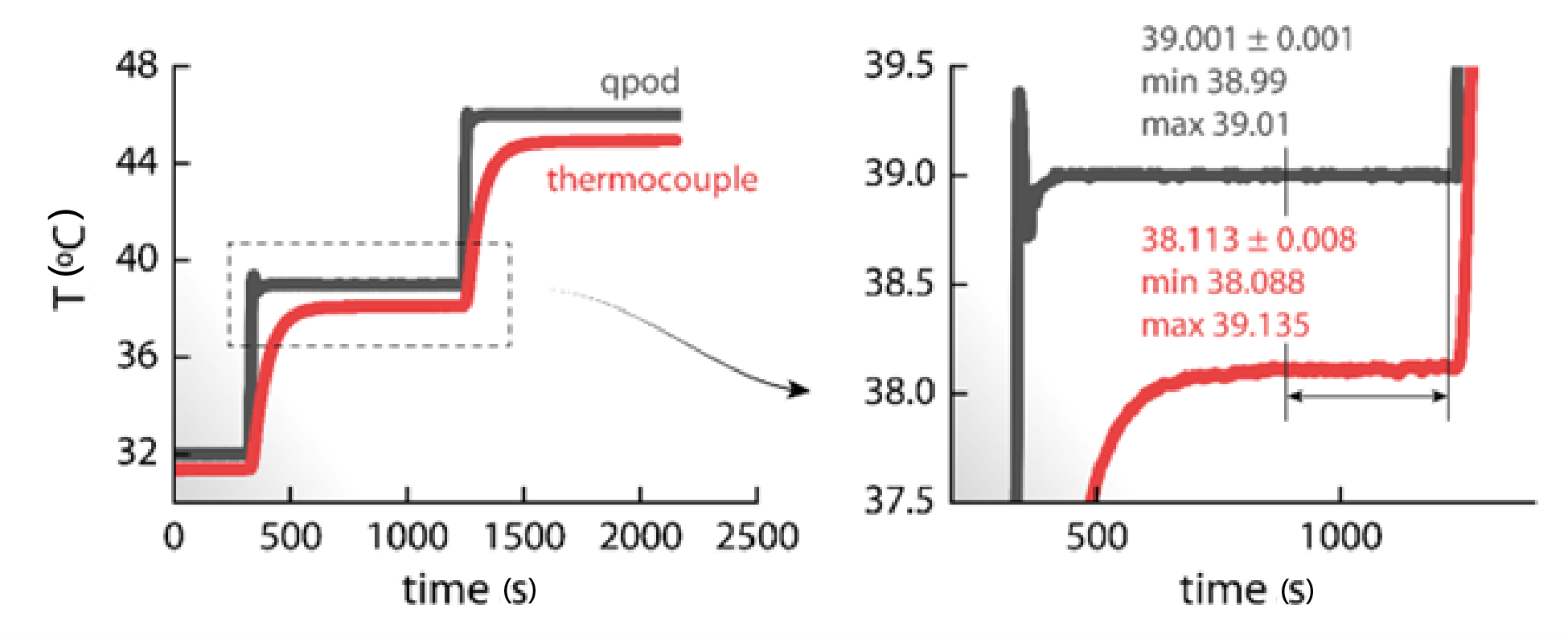


**Figure S15.** Temperature of the qpod (heating elements) and thermocouple readout at 3 temperatures (32, 39 and 46 ºC set on the temperature controller of the qpod). The numbers reported in the graph on the right were obtained from the analysis of the two curves in the interval indicted by the two vertical lines.
